# Neural crest derived progenitor cells contribute to tumor stroma and aggressiveness in stage 4/M neuroblastoma

**DOI:** 10.18632/oncotarget.21128

**Published:** 2017-09-21

**Authors:** Pedro Linares-Clemente, Diana Aguilar-Morante, Ismael Rodríguez-Prieto, Gema Ramírez, Carmen de Torres, Vicente Santamaría, Diego Pascual-Vaca, Ana Colmenero-Repiso, Francisco M. Vega, Jaume Mora, Rosa Cabello, Catalina Márquez, Eloy Rivas, Ricardo Pardal

**Affiliations:** ^1^ Instituto de Biomedicina de Sevilla (IBiS), Departamento de Fisiología Médica y Biofísica, Hospital Universitario Virgen del Rocío, CSIC, Universidad de Sevilla, Sevilla, Spain; ^2^ Departamento de Anatomía Patológica, Hospital Universitario Virgen del Rocío, Sevilla, Spain; ^3^ Departamento de Oncología Pediátrica, Hospital Universitario Virgen del Rocío, Sevilla, Spain; ^4^ Departamento de Cirugía Pediátrica, Hospital Universitario Virgen del Rocío, Sevilla, Spain; ^5^ Departamento de Oncología, Hospital Sant Joan de Déu, Barcelona, Spain

**Keywords:** neuroblastoma, neural crest-derived progenitors, angiogenesis, tumor stroma, smooth muscle actin (SMA)

## Abstract

Pediatric tumors arise upon oncogenic transformation of stem/progenitor cells during embryonic development. Given this scenario, the existence of non-tumorigenic stem cells included within the aberrant tumoral niche, with a potential role in tumor biology, is an intriguing and unstudied possibility. Here, we describe the presence and function of non-tumorigenic neural crest-derived progenitor cells in aggressive neuroblastoma (NB) tumors. These cells differentiate into neural crest typical mesectodermal derivatives, giving rise to tumor stroma and promoting proliferation and tumor aggressiveness. Furthermore, an analysis of gene expression profiles in stage 4/M NB revealed a neural crest stem cell (NCSC) gene signature that was associated to stromal phenotype and high probability of relapse. Thus, this NCSC gene expression signature could be used in prognosis to improve stratification of stage 4/M NB tumors. Our results might facilitate the design of new therapies by targeting NCSCs and their contribution to tumor stroma.

## INTRODUCTION

Neuroblastomas (NBs) are embryonic tumors arising during development from sympathoadrenal cells of the neural crest (NC) [1]. They are the most common extra-cranial solid tumors in children, being responsible for approximately 7–10% of pediatric cancers and 15% of all pediatric cancer deaths [2]. NBs are characterized by high heterogeneity within tumor phenotypes, ranging from spontaneous regression in stage 4S/MS low risk patients, to high-risk stage 4/M tumors that frequently present with metastasis and rapidly develop resistance to current treatments [1, 3]. In addition to the age at diagnosis, some genetic abnormalities have been described as prognostic for high risk NB, such as ploidy, amplification of MYCN oncogene, and gain or loss of some chromosomal regions [3].

In the past few years, the role of tumor stromal cells has emerged as a key point for the biology of several tumors [4]. Stromal cells have been described as important players in tumorigenesis, increasing tumor proliferation and angiogenesis [5], or selecting tumor cell clones responsible for distant metastasis [6–8]. Cancer associated fibroblasts (CAFs) are one of the most abundant components of tumor stroma [9]. Characterized by the expression of typical mesenchymal markers, such as SMA, Vimentin, Thy1 (CD90) or FSP1 (S100A4) [4, 9], the origin and nature of these cells is not well known. In NB, SMA positive CAFs are inversely correlated with histological features associated to low risk tumors [10]. Moreover, a gene signature characterized by the presence of mesenchyme related genes defines a subgroup of NBs with increased metastatic potential and poor prognosis [11]. This mesenchymal aggressive stroma should not be confounded with the neural schwannian stroma, also described in NB [10], which is rather based on schwann cells and associated to benign prognosis.

The NC is a transient embryonic multipotent cell compartment with the potential to differentiate into neural cells, but also into mesectodermal derivatives, such as chondrocytes, adipocytes, osteocytes and smooth muscle cells [12, 13]. Herein, we investigate whether NC progenitors could be found within aggressive NB tumor microenvironment, potentially contributing to tumor stroma formation by differentiating into mesectodermal derivatives, such as SMA positive cells. Our findings could help to understand a subset of aggressive NB tumors characterized by rich mesenchymal stroma and a neural crest gene-expression profile.

## RESULTS

### Isolation and characterization of primary cell cultures from NB tumor biopsies

**Figure 1:**
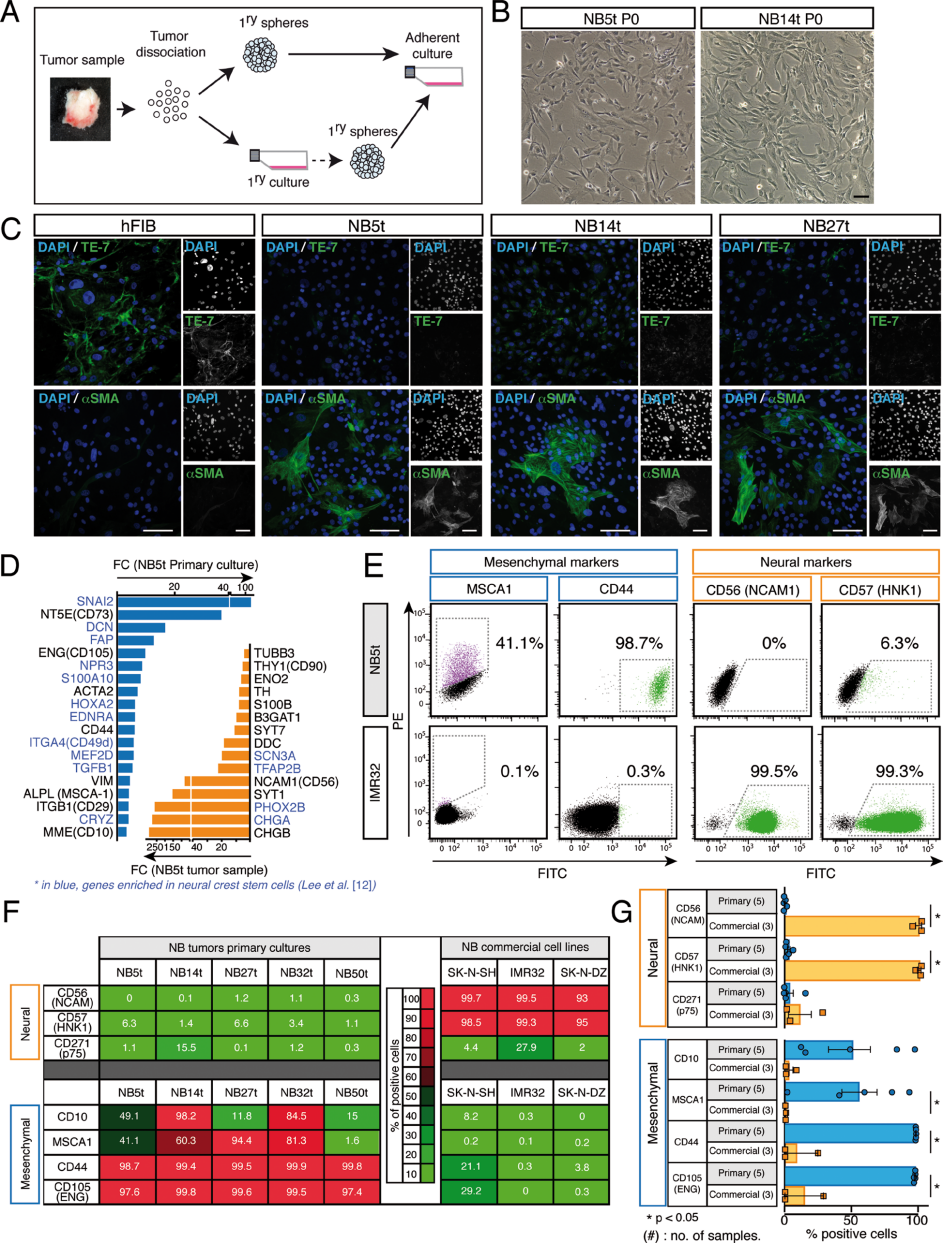
Characterization of stromal phenotype in primary adherent cell cultures isolated from stage 4/M NB tumor biopsies (**A**) Schematic view of cell isolation protocol from tumor biopsies. (**B**) Representative bright field images of primary adherent cells isolated from two different NB biopsies (NB5t and NB14t) showing the characteristic stromal-like phenotype of cultured cells. (**C**) Inmunofluorescent labeling of mesoderm-derived fibroblasts with TE-7 marker, or neural crest-derived myofibroblasts with smooth muscle actin (SMA) in a control human fibroblast cell line (hFIB) and in three different NB tumor-derived cell cultures (NB5t, NB14t and NB27t). (**D-G**) Primary cells expressed mesenchymal-like but not neuroblastic markers. (D) Gene expression analysis comparing a NB primary cell culture (blue bars) and its original tumor sample (orange bars) (NB5t). Genes were selected according to their relationship to mesectodermal or neuroblastic phenotypes and their reported enrichment in neural crest stem cells (see text for references). Based on this result, a panel of surface markers was selected to extend the gene expression analysis to 5 NB primary cell cultures and 3 NB commercial cell lines with a reported neuroblastic phenotype. (E) Representative flow cytometry plots showing expression of MSCA1, CD44 (mesenchymal markers), CD56 and CD57 (neuroblastic markers) in NB primary adherent cells from NB5t tumor and in the neuroblastic cell line IMR32. (F) Analysis of flow cytometry data showing the expression level (% of positive cells) for each surface marker tested in each NB tumor derived primary cell culture or in NB commercial cell lines. (**G**) Quantification of data shown in (F) (*p < 0.05, Mann-Whitney *U*-test), comparing primary cells versus cell lines. Scale bars in (B) and (C): 100 μm.

Aggressive NB tumor biopsies (Supplementary Table 1) were dissociated to single cells and cultured in ultra-low binding plates with neural crest medium [14], either directly or after a passage in adherent conditions (Figure 1A; see also Supplementary Figure 1). Resulting neurospheres were finally cultured in adherent conditions, again with neural crest specific medium (Supplementary Figure 1). As previously described [15], adherent conditions generally select for cells with stromal phenotype (Figure 1B). In contrast, neuroblasts only grew in suspension as spheres but tended to differentiate when attached to the substrate, disappearing from subsequent passages, indicating that our adherent primary culture protocol clearly favors the growth of stroma-like cells (see Supplementary Figure 1). On the other hand, the passage in low binding conditions (Supplementary Figure 1) is likely selecting for NS-forming NC-derived progenitor cells, which continue to grow after culturing onto adherent due to their stromal phenotype. Moreover, thanks to the neurosphere-forming passage, not any stromal cell found within tumors is present in our final adherent cultures. In fact, primary adherent cells obtained from tumor samples were negative for the expression of fibroblast specific antibody TE-7 [16], discarding the presence of patient derived-mesodermal fibroblasts (Figure 1C). Consistently, adherent cultures contained positive cells for smooth muscle actin alpha (aSMA), a typical NC marker that appears upon progenitor differentiation into mesenchymal lineages in adherent conditions [14, 17]. In addition, adherent primary cells expressed other typical NC markers such as p75/CD271, EDNRB, Nestin, GD2, CD44 or CD90 [18–20] (data not shown). Together, these results support the idea that our primary cultures are not simple mesodermal fibroblasts, but they rather consist of enriched NC-derived progenitor cells and their derivatives of mesectodermal lineage.

Next, we compared the expression profile of a tumor sample (NB5t) with its primary cells obtained in adherent conditions (Figure 1D). We focused on genes typical of mesenchymal or neural phenotypes, with special attention to genes enriched in neural crest stem cells (NCSCs) [13]. As expected, NB5t primary cells showed increased expression of mesenchymal genes (NT5E, ENG, ACTA2, MME, CD44, VIM or ALPL) [13, 18, 21, 22], while NB5t tumor sample expressed mostly neuronal genes (TH, ENO2, NCAM1, DDC or CHGB) [23–27] reflecting the neuroblastic phenotype of stage 4/M NB tumors. Genes over-expressed in NC progenitors were enriched in both samples, but with a clear difference between the two groups: NB5t primary cells showed high expression of NCSC genes related to the mesenchymal phenotype of the NC (such as S100A10, EDNRA or TGFB1) [28–30], while NB5t tumor sample was enriched in NCSC genes related to neural differentiation (PHOX2B and CHGA) [31, 32]. Based on these results, we extended this phenotypic analysis to 5 stage 4/M NB primary cell cultures (NB5t, NB14t, NB27t, NB32t, and NB50t) and 3 established NB commercial cell lines with neuroblastic phenotype (SK-N-SH, IMR32 and SK-N-DZ). Using flow cytometry, we determined the expression of surface markers typical of neuronal cells (CD56/NCAM1 and CD57/HNK1) [14, 24, 26], mesenchymal cells (CD10/MME, MSCA1/ALPL, CD44 and CD105/ENG) [13, 21, 22, 33], and neural crest stem cells (CD271/p75) [13] (Figure 1E–1G). Despite the variability observed between different samples, the results confirmed that primary adherent cells obtained from NB tumor biopsies, in contrast to commercial cell lines, present a stromal phenotype characterized by high expression of typical mesenchymal proteins and low expression of neuronal markers.

### NB primary cultures contain a subpopulation of neural crest progenitor cells

**Figure 2:**
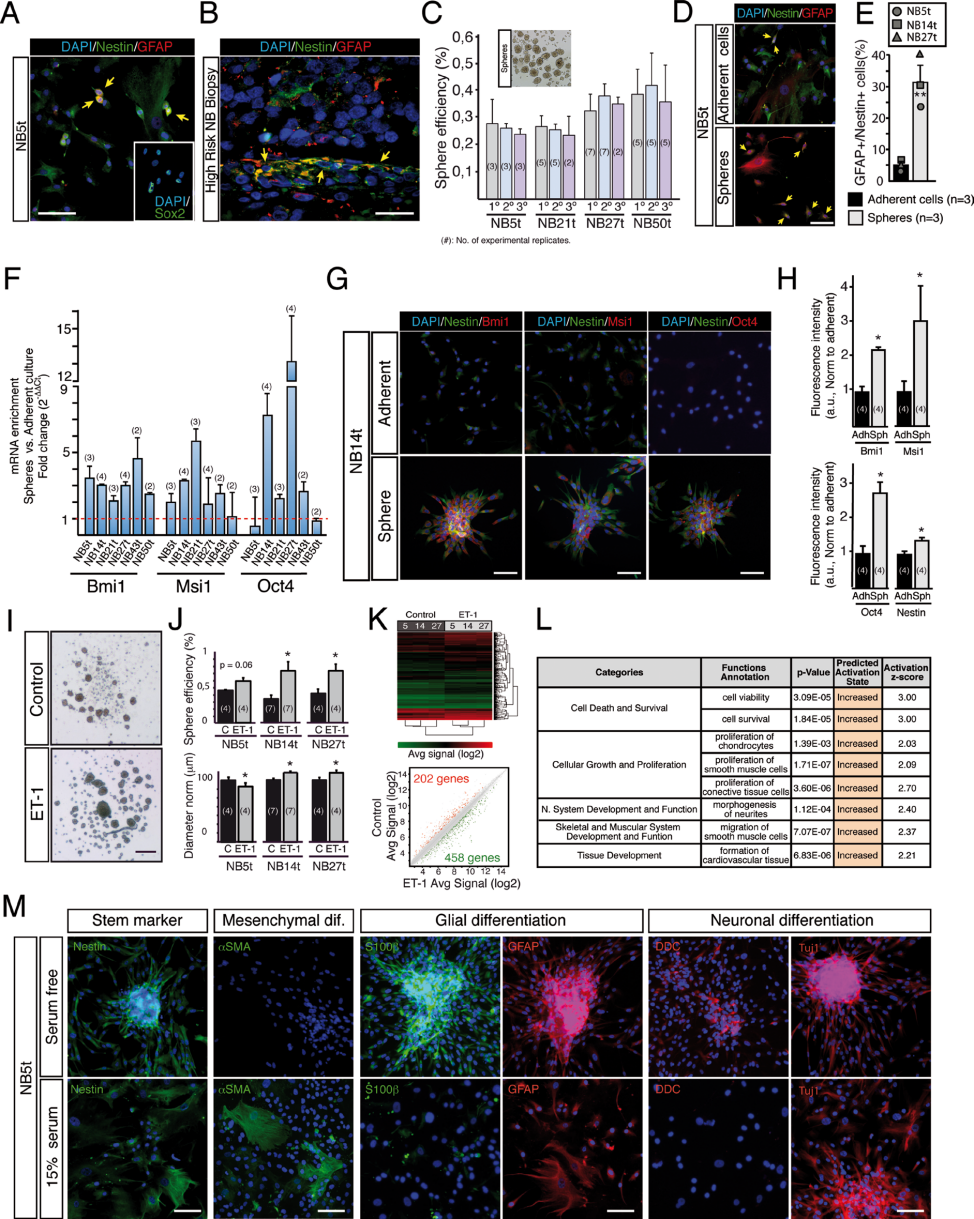
Stage 4/M NB tumor-derived primary cultures contain a subpopulation of neural crest progenitor cells (**A**) Representative photomicrograph showing nuclei (DAPI; blue), Nestin (green) and GFAP (red) stainings in a NB tumor derived primary adherent culture. Nestin/GFAP double positive cells are pointed with yellow arrows. Inset: Expression of Sox2 (green) in NB5t primary adherent cells. Scale bar: 100 μm. (**B**) Representative picture showing the existence of GFAP/Nestin double positive cells (yellow arrows) in an original high-risk NB tumor tissue. Scale bar: 25 μm. (**C**) Primary cultures contain a subpopulation of cells that grow as spheres when cultured in non-adherent conditions. The bright field image on top shows typical spheres formed when NB tumor-derived adherent primary cells were cultured in low-binding conditions. Graph quantifies sphere-forming efficiency from 4 different tumor-derived samples, measured in primary, secondary and tertiary sphere passages, revealing the existence of a small but self-renewing fraction of sphere-forming progenitor cells. (**D**) Immunocytochemistry showing nuclei (DAPI; blue), Nestin (green) and GFAP (red) expression in cells from adherent cultures and from spheres grown in parallel. Nestin/GFAP double positive cells are pointed with yellow arrows. Scale bar: 100 μm. (**E**) Quantification of GFAP/Nestin double positive cells from 3 different primary cultures (NB5t, NB14t and NB27t) and their corresponding spheres. In general, spheres showed a clear increase in the percentage of double positive cells (from 5% to 31%) (**p < 0.01, Student's *t*-test). (**F–H**) Spheres are enriched in genes described as neural crest stem cell markers when compared to adherent cell cultures. (F) Quantitative PCRs showing a clear increment in the expression of Bmi1, Msi1 and Oct4 mRNAs (neural crest stem cell markers) in spheres when compared to their corresponding adherent cultures (level 1 line). The variability observed could be explained by tumor heterogeneity between patients. (G, H) Increase in mRNA expression was confirmed at protein level in primary cell cultures from tumor biopsy NB14t. (G) Immunohistochemistry showing the expression of Nestin (green) and Bmi1, Msi1 or Oct4 (red) in primary cell adherent cultures and spheres grown in parallel. Scale bars: 100 μm. (H) Quantification of expression levels based on the intensity of fluorescence (arbitrary units normalized to adherent) confirmed the increase in neural crest stem cell marker mRNA expression shown in (F) (*p < 0.05, Student's *t*-test). (**I–L**) Endothelin-1 (ET-1) increases survival and proliferation of neural crest progenitors within primary cultures. (I) Representative bright field images of spheres cultured in control conditions or after ET-1 treatment. (J) Top. Quantification of sphere-forming cell frequency illustrating how ET-1 increases the % of these cells in all primary cultures tested. Bottom. Quantification of sphere diameter showing how two of the three primary cell cultures also present a small but significant increase in sphere diameter when treated with ET-1. Both analyses together suggest an increase in survival and proliferation of neural crest progenitors treated with ET-1. (K, L) ET-1 mediated effect was confirmed by gene expression profiling of control and ET-1 treated spheres from three different primary cell cultures. (K) Hierarchical clustering and scatter plot obtained from a microarray gene expression analysis performed to compare control versus ET-1 treated spheres. (L) Ingenuity Pathway Analysis of genes differentially expressed (660 genes) after ET-1 treatment, predicting an increase in biofunctions like cell viability and cell survival, as well as in functions fully compatible with a neural crest origin of the spheres analyzed. (**M**) Immunofluorescent stainings of NB tumor-derived spheres after 4–5 days of differentiation in adherent conditions, with or without 15% serum. Cells were labeled with antibodies against nestin (stem cell marker), SMA (mesenchymal differentiation marker), S100b and GFAP (glial differentiation markers), and DDC and Tuj1 (neuronal differentiation markers). Scale bars: 100 μm.

NC derived progenitors can be found in postnatal tissues, and are characterized by the expression of the glial fibrillary acidic protein (GFAP) [14, 17]. NB primary adherent cultures presented cells double positive for GFAP and Nestin, an intermediate filament expressed in proliferating neural stem cells [34] (Figure 2A). Consistently, most adherent cells also expressed the neural stem cell typical transcription factor Sox2 [35] (inset in Figure 2A). The GFAP/Nestin double positive phenotype was also found, at a reasonable frequency (1.2 ± 0.3%; *n* = 14 tumor samples), in the original tumor tissue (Figure 2B). To confirm the existence of NC derived progenitors in NB primary cultures, cells were challenged to form spheres in low binding substrate [36]. All NB samples tested formed spheres that were able to self-renew and that showed a remarkable increase in the percentage of GFAP/Nestin double positive cells (Figure 2C–2E). Moreover, spheres showed a clear increase in the expression of genes typical for NC progenitors, such as BMI1, MSI1 and OCT4 [37, 38] (Figure 2F–2H), indicating a clear enrichment in NC-derived progenitor cells.

We next determined the phenotype of proliferative cells in NB primary cultures. 74.8 ± 6.7% of all cells undergoing mitosis in adherent conditions (identified by the presence of mitotic figures; Supplementary Figure 2) were positive for GFAP and Nestin, indicating a major role of double positive progenitor cells in proliferation and growth of primary cultures. In addition, we observed putative asymmetric cell divisions within the proliferating population, an evolutionary conserved division mode used by stem and progenitor cells [39] (see Supplementary Figure 2).

### Endothelin-1 increases survival and proliferation of neural crest progenitors

Endothelin-1 (ET1) is a cytokine that regulates proliferation, migration, differentiation and survival of NC cells [19, 40]. Moreover, our group has previously shown a strong positive effect of ET1 on the proliferation of adult neural crest stem cells in the carotid body [41]. The addition of ET1 increased the number and diameter of spheres obtained from NB primary cultures, compatible with an increase in survival and proliferation of progenitor cells (Figure 2I, 2J, and Supplementary Figure 3). Gene expression profiling of spheres cultured from three different adherent primary cell samples revealed that ET1 treatment induced changes in the expression of 660 genes, from which 458 were up regulated. As expected, Ingenuity Pathway Analysis of differentially expressed genes revealed an increase in bio-functions related to cell survival, viability and proliferation. Interestingly, the analysis also uncovered functions assigned to cell types closely related to the NC, such as chondrocytes, smooth muscle, neurons or cardiovascular tissues (Figure 2K, 2L). Altogether, sensitivity to ET-1 and gene expression profiling analysis support a neural crest-derived stem cell phenotype in NS-forming progenitor cells cultured from NB adherent primary cells.

### Neural crest progenitors can differentiate into both neural and mesectodermal lineages

The NC is a multipotent embryonic structure with the potential to differentiate to neural cells, but also to mesectodermal derivatives [12]. In addition, our group has recently described that neural crest progenitors in the adult carotid body retain this multipotentiality [42, 43]. To further characterize NB derived primary cells, we tested the differentiation capacity of these cells *in vitro* (Figure 2M, and Supplementary Figure 4) using different serum conditions (see Methods). Staining with both neural and mesenchymal markers revealed that NB spheres contained progenitor cells that were able to differentiate into neural cells (positive for GFAP, S100b, DDC or Tuj1), but also into typical mesenchymal-like derivatives, with a remarkable expression of SMA, a marker widely used to label cancer associated fibroblasts [4, 9]. Altogether, our results are fully compatible with the existence of a subpopulation of neural crest derived progenitor cells in NB tumor biopsies. These progenitors generate primary cell cultures with characteristic mesectodermal stromal phenotype.

### Neural crest progenitors isolated from NB biopsies are not tumorigenic

At this point, we wondered whether these neural crest progenitor cells behaved as cancer stem cells, being tumorigenic and able to recapitulate patient tumor formation in immunocompromised mice. Cells from six different primary cultures were xenografted both subcutaneously and orthotopically (in the adrenal medulla) of immunosuppressed mice. Surprisingly, none of the mice developed tumors (Supplementary Table 2), despite the highly efficient tumorigenesis exhibited in the same assay by an IMR32 cell line positive control. Genomic analysis of these NB primary stromal cells revealed the absence of NB characteristic genomic alterations, such as MYCN amplification (sample NB5t), as compared to original tumors. Multiplex Ligation-dependent Probe Amplification (MLPA) analysis confirmed that NB primary adherent cells lacked some of the chromosomal aberrations present in tumor biopsies (Supplementary Figure 5). These results confirmed that, despite their neural crest origin, these NB tumor-derived progenitor cells lack critical genomic alterations, which could explain the absence of tumorigenicity.

### NB primary stromal cells increase proliferation of NB cell lines *in vitro* and promote tumor growth in a xenograft model *in vivo*

**Figure 3:**
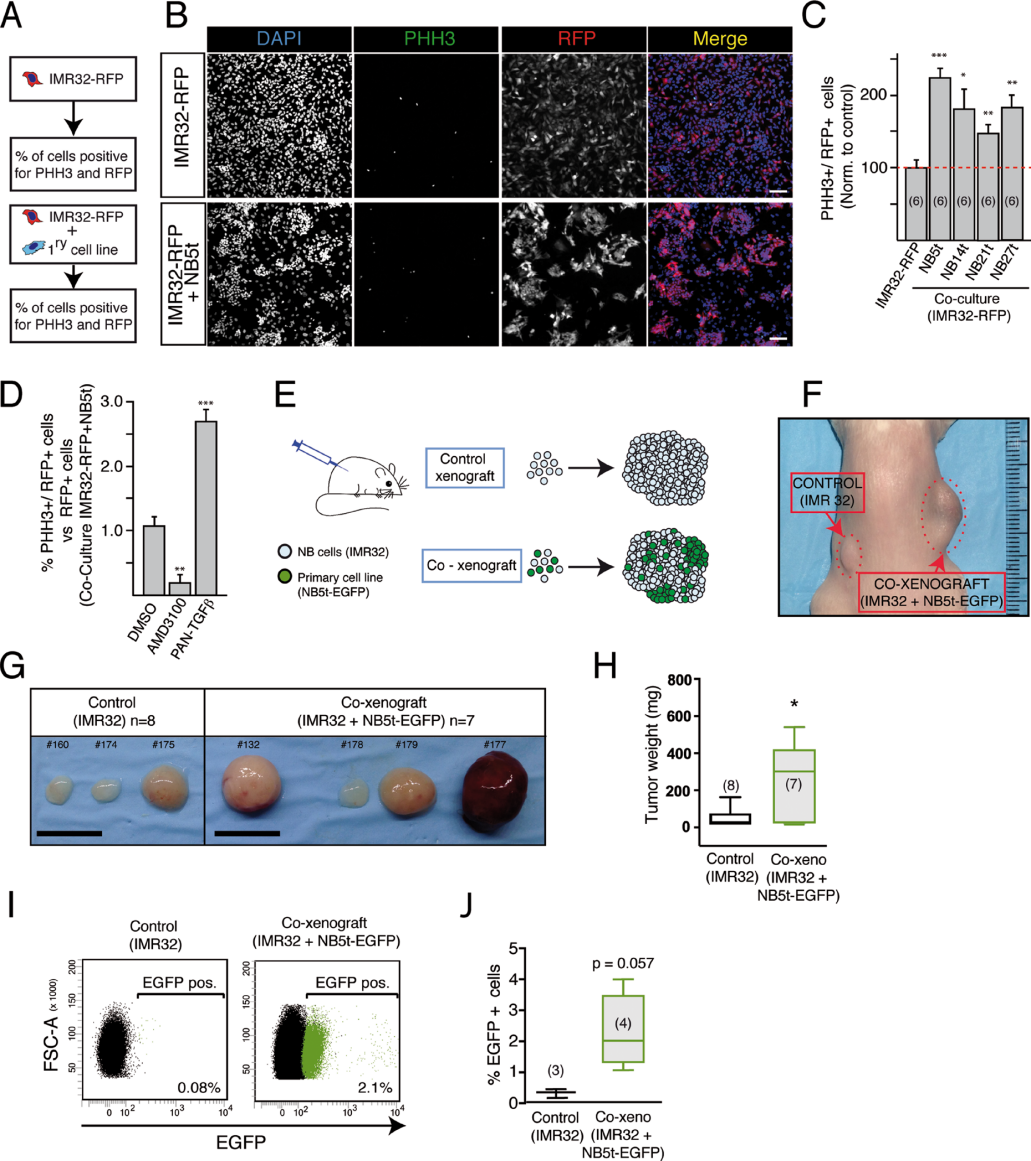
NB tumor-derived adherent cells increase proliferation of NB cell lines *in vitro* and favor tumor growth *in vivo* (**A**) Diagram of the experimental design of *in vitro* co-cultures. IMR32 NB cells expressing a red fluorescent protein (IMR32-RFP) were cultured alone or with NB tumor-derived adherent cells at 1:1 ratio. After 4 days in culture, the % of IMR32-RFP proliferative cells (PHH3+) was measured. (**B**) Representative fluorescent images of cultures showing nuclei (blue), proliferative cells (PHH3+; green) and IMR32-RFP cells (red fluorescent protein+; red). Scale bars: 100 μm. (**C**) Quantification of the % of PHH3+ IMR32-RFP cells in control (IMR32-RFP cells cultured alone) and in co-cultures with adherent cells derived from 4 different NB tumors (NB5t, NB14t, NB21t and NB27t). Results are normalized to the % of PHH3+ cells in control cultures (*p < 0.05; **p < 0.01; ***p < 0.001, Student's *t*-test). (**D**) Quantification of IMR32-RFP proliferative cells (PHH3+) in co-cultures with NB5t cells, in the presence of AMD3100 and PAN-TGFβ inhibitors, always compared to control co-culture in the presence of vehicle (DMSO) (**p < 0.01; ***p < 0.001, Student's *t*-test). (**E**) Experimental design of co-xenografts. (**F**) Representative image of a mouse xenografted with IMR32 cells alone (control xenograft, left flank) or admixed with NB5t derived adherent cells (co-xenograft, right flank). (**G**) Examples of different tumors after 2 months of growth, illustrating the bigger sized phenotype of co-xenografts. Scale bars: 10 μm. (**H**) Quantification of tumor weight in control and co-xenograft tumors. (*p < 0.05, Mann-Withney *U*-Test). (**I**) Representative flow cytometry plots showing the incorporation of a small but consistent % of EGFP positive NB derived stromal cells in co-xenograft tumors. (**J**) Quantification of EGFP positive cells (%) in control and co-xenograft tumors (*p* = 0.057, Mann-Whitney *U*-Test).

We next asked whether these tumor-derived neural crest progenitor cells could affect the biology and behavior of other NB tumor cells. We performed co-cultures of IMR32 commercial cell line with or without cells from four different primary cell cultures (NB5t, NB14t, NB21t, and NB27t). In all cases, co-cultures increased proliferation of IMR32 cells (Figure 3A–3C). In order to have a glimpse of the type of paracrine signaling being established between primary cells and tumorigenic IMR32 cells, we repeated the co-culture experiments in the presence of specific inhibitors (Figure 3D). Stromal cells typically secrete both SDF-1 and TGFβ cytokines. AMD3100 is an inhibitor of CXCR4 receptor [44], which has been involved in malignant progression of NB through SDF-1 signaling [45], and PAN-TGFβ is an antibody that blocks TGFβ signaling [46]. Our results preliminarily suggest that SDF-1/CXCR4 signaling is important when promoting IMR32 proliferation, while TGFβ signaling seems to inhibit this proliferation (Figure 3D). These signaling routes constitute interesting examples of how primary adherent stromal cells might be regulating the aggressive behavior of IMR32 cells *in vitro*.

**Figure 4:**
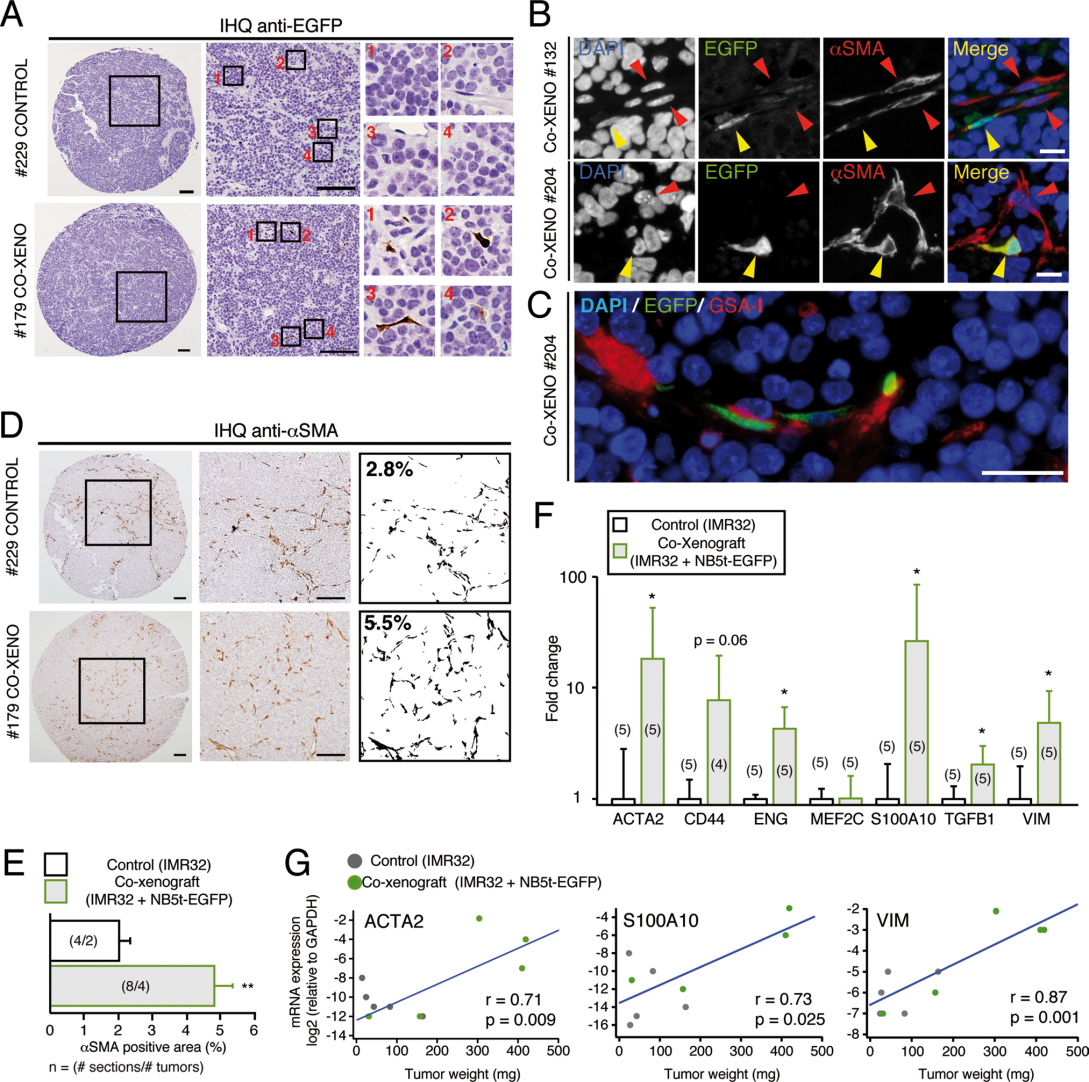
NB tumor-derived adherent cells give rise to SMA+ perivascular stromal cells *in vivo* (**A**) Representative images of anti-EGFP immunohistochemistry in control and co-xenograft tumors. Tumors supplemented with primary adherent cells (lower panels) show EGFP positive cells (insets 1–4) with characteristic stromal cell morphology. Scale bars: 100 μm. (**B**) Immunofluorescent images detecting nuclei (blue), EGFP (green) and SMA (red) in two different co-xenografts. EGFP+ cells differentiate into SMA positive cells (yellow arrowheads) usually associated to other SMA+ cells (red arrowheads). Some of the double positive cells show characteristic perivascular localization (Co-XENO #132). Scale bars: 10 μm. (**C**) Representative immunofluorescent picture of a co-xenograft tumor detecting EGFP (green) and an endothelial cell-specific lectin (GSA I; red), illustrating the typical perivascular localization of primary adherent cells. Scale bar: 50 μm. (**D**) Immunohistochemistry against SMA in sections of control and co-xenograft tumors. Images were binarized (right panels) and the % of SMA positive area quantified. Scale bars: 100 μm. (**E**) Quantification of the % of SMA positive area in control and co-xenograft tumors (**p < 0.01, Mann-Whitney *U*-Test). (**F, G**) Tumor supplementation with NB5t derived stromal cells increase the expression of mesenchymal-like genes. (F) Quantitative PCR analysis of the expression of mesenchymal genes in control and co-xenograft tumors. Expression levels of ACTA2 (encoding for SMA), CD44, ENG, MEF2C, S100A10, TFGB1 and VIM mRNAs are shown normalized to their expression in control tumors (*p < 0.05, Student's *t*-test). (G) Correlation analysis of tumor weight (of both control and co-xenografts) versus mRNA expression levels of ACTA2, S100A10 and VIM (relative to GAPDH) (r = Pearson's correlation coefficient). These correlations indicate that the higher the expression of mesenchymal markers the bigger the tumor size.

The co-culture results obtained *in vitro* prompted us to investigate a potential role of these NC progenitor-derived stromal cells in promoting tumorigenesis *in vivo*. IMR32 cells were injected subcutaneously alone or with EGFP expressing primary cells from NB5t sample at a 1:1 ratio (Figure 3E). Tumor supplementation with NC derived stromal cells caused a significant increase in tumor size (Figure 3F–3H). Flow cytometry (Figure 3I, 3J) and immunohistochemistry (Figure 4A) confirmed that EGFP expressing NC derived primary cells were incorporated into tumor structure. Almost 100% of EGFP positive cells within tumor parenchyma were positive for SMA (Figure 4B), and showed characteristic perivascular localization (Figure 4C). In addition, immunohistochemistry analysis revealed that NB primary cell supplementation caused a significant increase in SMA expression (Figure 4D, 4E), confirmed by qPCR (Figure 4F). In addition to ACTA2 (SMA), expression analysis showed a significant increase in genes related to a mesenchymal phenotype, such as CD44, ENG, S100A10, TGFB1 and VIM (Figure 4F). Interestingly, expression levels of ACTA2, S100A10 and VIM were positively and significantly correlated to tumor size, suggesting a relationship between NC derived stromal component and tumor growth (Figure 4G).

### Neural crest stem cell gene signature is associated to increased aggressiveness in stage 4/M neuroblastoma

**Figure 5:**
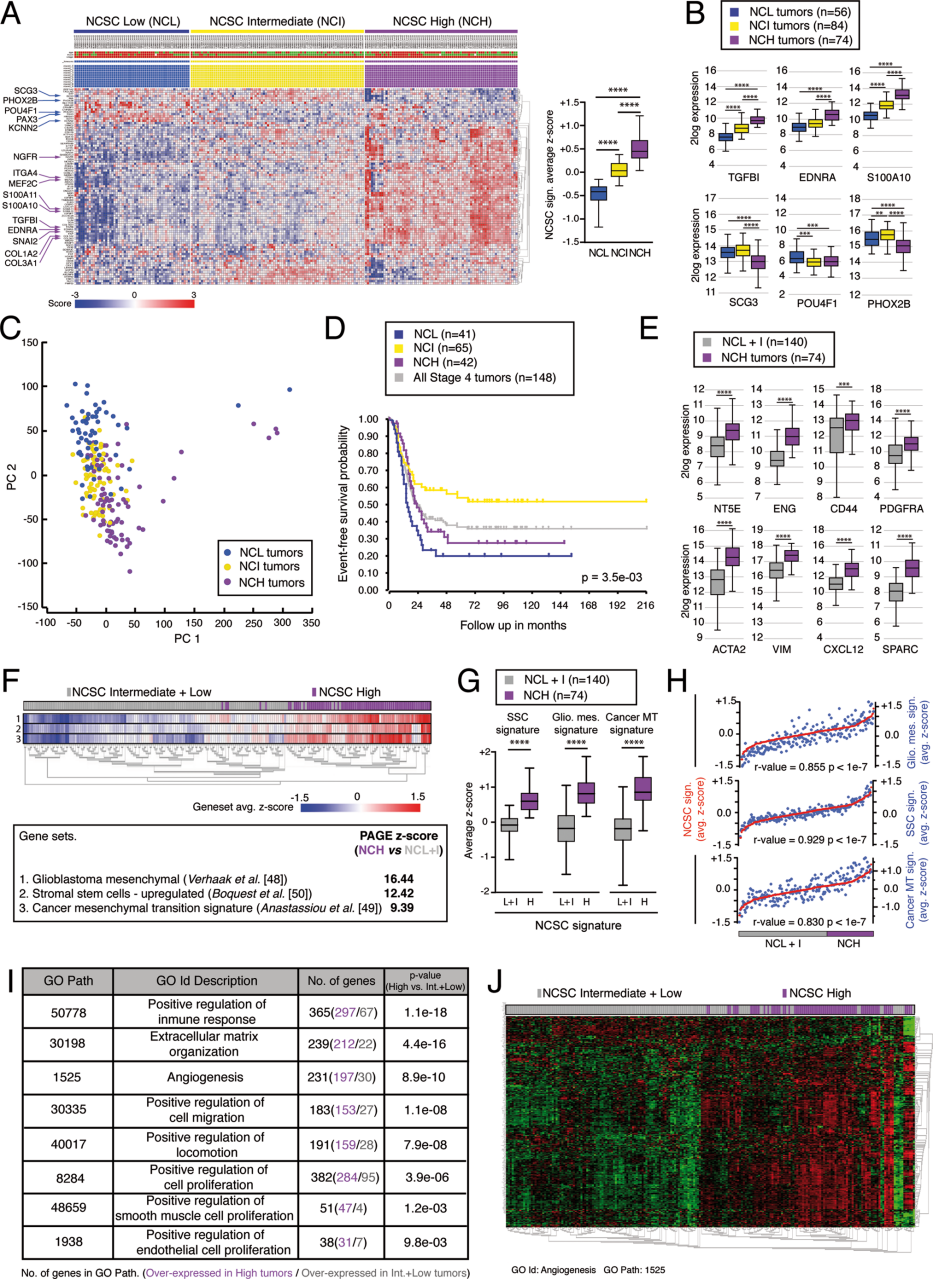
A neural crest stem cell (NCSC) gene signature segregates stage 4/M NB tumors and is associated to tumor aggressiveness (**A-D**) NCSC gene signature defines a subgroup of stage 4/M NB tumors with high probability of relapse. (A) K-means clustering using genes up-regulated in NCSCs (described in *Lee et al.* [13]), segregating stage 4/M NB tumors into three different groups depending on high, intermediate or low expression levels of the signature (NCH, NCI and NCL tumors respectively). (B) NCH tumors are enriched in NCSC genes related to the mesenchymal phenotype of the neural crest, such as TGFB1, EDNRA and S100A10. By contrast, genes related to neural specification of NCSCs, such as SCG3, POU4F1 and PHOX2B, show lower expression levels within NCH tumors. (C) Transcriptome wide profiling by principal component analysis, revealing a clear segregation between NCH, NCI and NCL tumors. Principal component 2 (PC2) maximized the differences between the three types of tumors. (D) Kaplan-Meier curves showing event-free survival probability of NCH, NCI and NCL tumors, compared to all stage 4/M tumors analyzed together. NCH and NCL tumors present higher probability of relapse compared to NCI neuroblastomas. (**E-H**) NCH express higher level of mesenchymal related genes. (E) Expression levels of typical mesenchymal genes in NCH tumors compared to NCI and NCL tumors together (NCL+I). All genes analyzed (NT5E, ENG, CD44, PDGFRA, ACTA2, VIM, CSCL12 and SPARC) showed higher expression levels in NCH tumors than in NCL+I tumors. (F) Parametric analysis of gene set enrichment (PAGE) in NCH tumors revealed enrichment in the expression of genes previously described as characteristic of a mesenchymal phenotype. Enrichment z-score for three different gene sets is shown: genes typical of the mesenchymal subtype in glioblastoma (gene set #1, described in *Verhaak et al.* [48]), genes up-regulated in stromal stem cells (gene set #2, described in *Boquest et al.* [50]), and genes typical of mesenchymal transition in cancer (gene set #3, described in *Anastassiou et al*. [49]). The heat map in the upper panel shows the complete gene set map of average z-scores for all genes in every single gene set, calculated for each individual sample. (G) Box-whiskers plots showing the expression of the three different gene sets analyzed in NCH, but now compared to NCL+I tumors. (H) Correlation between gene set enrichment average z-scores in individual tumors over the whole sample of tumors analyzed. NCSC z-scores were correlated to enrichment z-scores for the gene sets analyzed in (F). The results revealed a close correlation between expression of genes up-regulated in NCSCs and genes related to mesenchymal features, both being highly expressed in NCH tumors. (**I, J**) Genes differentially expressed in NCH tumors are involved in biofunctions and processes related to the stromal component of tumors. (I) Representative biofunctions and processes resulting from gene ontology analysis of genes differentially expressed between NCH and NCL+I tumors. Genes overexpressed in NCH tumors participate preferentially in processes related to the stromal component of tumors, such as “positive regulation of immune response”, “extracellular matrix organization” and “angiogenesis”. (J) Heat map showing the expression levels of genes involved in angiogenesis (GO Path 1525). Tumors are hierarchically clustered and NCSC enrichment status is shown in the upper bar. Whiskers in Box-Whiskers plots indicate maximum and minimum values. (r-value = Pearson's correlation coefficient) (****p < 10^–4^, ***p < 0.001, **p < 0.01; Mann-Whitney *U*-test).

The results obtained led us to consider the existence of a specific neural crest stem cell (NCSC) genetic profile linked to mesenchymal phenotype and poor prognosis in stage 4/M NB. A set of 214 stage 4/M tumors [47] was analyzed *in silico* (see Methods). K-mean clustering based on the expression of genes up-regulated in NCSCs [13] generated three different groups with high, intermediate and low expression of NCSC gene signature (NCH, NCI and NCL respectively) (Figure 5A, 5B). The analysis of transcriptome-wide profiles by principal component analysis confirmed the existence of clear differences between these three groups, with a maximum segregation between NCH and NCL tumors through principal component 2 (Figure 5C). In addition, NCH and NCL tumors showed increased aggressiveness and high probability of relapse when compared to NCI (Figure 5D). Next, we focused on NCH tumors and analyzed the expression of typical mesenchymal genes such as NT5E (CD73), ENG, CD44, PDGFRA, ACTA2 (SMA), VIM, CXCL12 or SPARC. The levels of all these mesenchymal genes were significantly increased in NCH tumors when compared to NCL and NCI tumors together (NCL+I for brevity) (Figure 5E). Moreover, we carried out a parametric analysis of gene set enrichment (PAGE) comparing the expression of known gene set profiles in NCH versus NCL+I tumors. Interestingly, within the most highly enriched gene sets in NCH tumors, we found gene signatures typical of the mesenchymal subtype in glioblastoma [48] and the mesenchymal transition signature associated to cancer [49] (Figure 5F–5H). In addition, compatible with an increase in NCSC genes related to the mesenchymal phenotype, we also detected enrichment in genes up regulated in human stromal stem cells (SSCs; Figure 5F–5H) [50]. Next, we wondered about the putative role of such a mesenchymal component in increasing tumor aggressiveness. To try to solve this question, we determined the genes that were differentially expressed between NCH and NCL+I tumors and performed a gene ontology analysis to reveal the biological significance of the different expression profiles. Interestingly, NCH tumors showed a significant enrichment in biofunctions like “cell proliferation”, “migration” and cellular processes related to the stromal component of tumors [4], such as “immune response”, “extracellular matrix organization”, “proliferation of endothelial cells”, “proliferation of smooth muscle cells” and “angiogenesis” (Figure 5I, 5J). Altogether, this *in silico* analysis suggests that a neural crest-derived progenitor component within aggressive NB tumors promotes the formation of mesectodermal stroma, with subsequent facilitation of angiogenesis and other processes necessary for tumor growth.

### Expression of SMA correlates with NCSC gene signature and is associated to histopathological features of aggressive neuroblastomas

**Figure 6:**
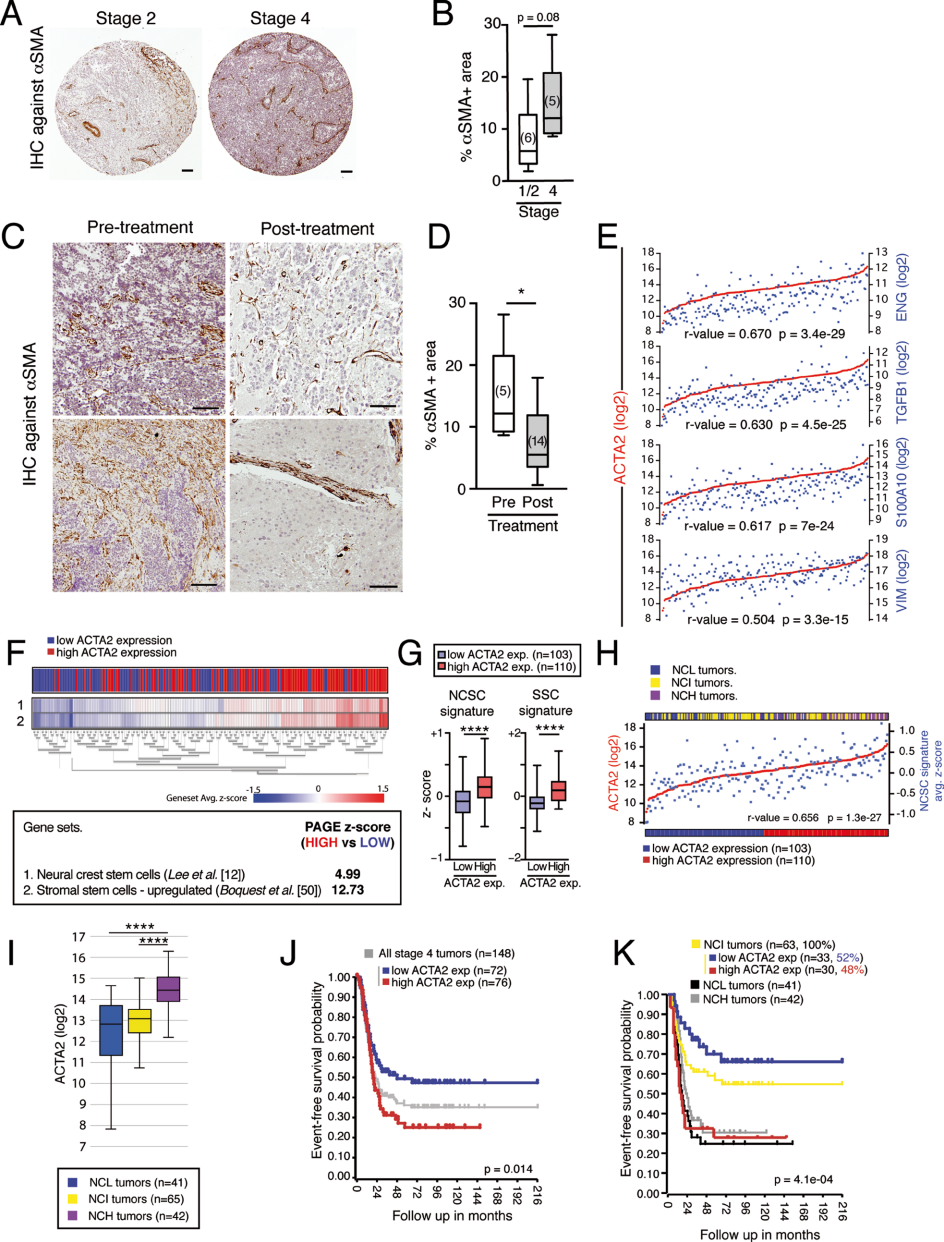
SMA correlates to NCSC gene signature expression and aggressiveness in stage 4/M NBs (**A-D**) Association between SMA protein expression and histopathological features typical of stage 4/M NBs. (A) Representative images of anti-SMA immunohistochemistry in sections from Stage 1–2 vs. Stage 4 tumors. Scale bars: 100 μm. (B) Expression of SMA (% of stained area) was calculated as in Figure 4E. Aggressive neuroblastomas (Stage 4/M) showed higher expression levels than low-risk tumors (Stage 1 and 2). (C) Representative images of anti-SMA immunohistochemistry in stage 4/M tumor sections before and after treatment. Scale bars: 100 μm. (D) Quantification of SMA expression (% of stained area) in pre- and post-treatment tumor sections. Concomitantly to treatment-induced tumor differentiation (considered as an indicator of treatment response), we observed a significant decrease in SMA expression levels. (**E**) ACTA2 (gene encoding for SMA) expression correlates with mesenchymal genes (ENG, TGFB1, S100A10 and VIM). (**F**) Parametric analysis of gene set enrichment (PAGE) revealed that tumors with higher expression of ACTA2 were also enriched in expression of typical NCSC genes (gene set #1, described in *Lee et al*. [13]) and MSC genes (gene set #2, described in *Boquest et al*. [50]). Heat map (upper panel) shows the average z-scores for all genes in every single gene set, calculated for each individual tumor sample. (**G**) Quantification of the expression of NCSC or MSC gene signatures in ACTA2 high compared to ACTA2 low tumors. (**H**) Correlation between ACTA2 expression and NCSC gene signature average z-scores over the whole sample of tumors analyzed. (**I**) Tumors enriched in expression of NCSC genes (NCH) show higher expression levels of ACTA2. (**J**) Kaplan-Meier curves showing the association between high ACTA2 expression levels and lower event-free survival probability. (**K**) Kaplan-Meier curves showing the event-free survival probability of NCI tumors segregated into ACTA2 high (48%) and ACTA2 low (52%) tumors. (r-value = Pearson's correlation coefficient) (****p < 10^–4^, *p < 0.05; Mann-Whitney *U*-test). See also Supplementary Figure 6.

SMA is a typical marker of cancer-associated fibroblasts (CAFs), one of the most abundant components of tumor stroma [4, 9]. Immunohistochemistry of patient derived tumor samples (Supplementary Table 3) revealed a clear association between SMA expression (measured as % of SMA positive area) and histopathological features of aggressive tumors and bad prognosis (Figure 6A, 6B). Moreover, since chemotherapy treatment is associated to an acquired benign histopathology [51], post-treatment tumors exhibit a decrease in SMA expression (Figure 6C, 6D). *In silico* analysis of 214 stage 4/M tumors [47] showed a high level of correlation between the expression of ACTA2 (SMA) and other mesenchymal genes such as ENG, TGFB1, S100A10 and VIM (Figure 6E). Based on the ability of NC progenitor cells to give rise to SMA positive mesectodermal derivatives [12, 13], we wondered whether ACTA2 expression could be used as a predictor of NCSC gene signature enrichment in NB tumors. PAGE analysis revealed that tumors with high expression of ACTA2 were enriched in NCSC genes, as well as in stromal stem cell genes (Figure 6F, 6G). In addition, ACTA2 expression and NCSC signature enrichment average z-scores showed a positive correlation (Figure 6H, 6I). Moreover, the expression level of ACTA2 was significantly associated with poor prognosis (Figure 6J), revealing again a close relationship between mesenchymal features, NCSC signature enrichment and tumor aggressiveness. Combining tumor segregation based on NCSC gene signature enrichment and ACTA2 expression level, we observed that most NCH tumors (86%) express high levels of ACTA2, while most NCL samples (76%) belong to the ACTA2 low expression group, which is in agreement with the role of NCSCs giving rise to ACTA2 expressing stromal cells. Interestingly, SMA expression segregates NCSC Intermediate tumors (NCI) into two groups with different event-free survival probability (Figure 6K). ACTA2 highly expressing NCI tumors (48%) show a low event-free survival probability, similar to tumors belonging to NCH and NCL groups, when compared to ACTA2 low expressing NCI tumors (52%). Together, these results support the interest of SMA staining and ACTA2 expression levels as prognostic markers associated to NC derived stromal content in NB tumors. Therefore, combination of these two analyses, NC gene signature and ACTA2 expression, results in a highly accurate stratification of stage 4/M tumors in terms of aggressiveness.

## DISCUSSION

In the present study, we describe the existence of neural crest (NC) derived stem-like cells in aggressive neuroblastoma (NB) primary tumors. Cancer stem cells (CSCs) have become a major focus in cancer research for the last decade. Such an interest lies in the ability of these cells to initiate and propagate tumors, as well as their resistance to conventional treatments [52, 53]. NBs are embryonic tumors arising from cells belonging to the sympathoadrenal lineage of the NC [1]. Existing data suggest that malignant transformation can occur at any developmental stage of sympathoadrenal neural crest progress [54]. Consistently, CSC-like populations have been described in NB tumor samples and cell lines, presenting expression of stem-like genes and high tumorigenicity [55, 56]. NC progenitor cells described in our work are enriched in genes previously described in NC stem cells, such as BMI1, OCT4 and MSI1 [37, 38]. Moreover, these NC progenitor cells can differentiate into both neural (positive for GFAP, S100b, DDC or Tuj1) and mesectodermal cells (positive for CD105, CD44, MSCA1 or SMA) *in vitro*. However, the absence of tumorigenicity under our experimental conditions rule out the possibility of considering these cells similar to previously described cell populations of NB CSCs. Instead, our culture conditions selected for mesenchymal-like cells lacking critical genomic alterations when compared to their original tumor biopsies. Our results are compatible with the isolation of non-tumorigenic undifferentiated NC progenitor cells. These cells divide and give rise to stromal-like cells when cultured *in vitro*, contributing to tumor stroma formation and aggressiveness in a mouse model of NB. Future research should determine how general is this phenomenon of non-tumorigenic stem/progenitor cells being included in tumors [57], and should study the role of such cell population in tumor biology.

During the last years, tumor stroma has emerged as a key player in the biology of most types of cancers. In addition to highly proliferative tumorigenic cells, tumors are composed by a variety of non-tumorigenic cell types such as endothelial cells, pericytes, immune cells or cancer-associated fibroblasts (CAFs). Together with the extracellular matrix, these cells constitute the tumor microenvironment and promote aggressiveness, participating in processes such as tumor growth, metastasis, inflammation or angiogenesis [4–9].

Since NC stem cell populations have been described in several adult neural crest derived tissues [12], a likely explanation to our results could be that some NC progenitors get confined to the aberrant niche being orchestrated by transformed neuroblasts during NB tumor formation. These NC progenitors could easily contribute to tumor stroma due to their capacity to give rise to NC-typical mesectodermal derivatives. Consistent with this, we demonstrate that supplementation with NC derived stromal cells promotes proliferation and tumor aggressiveness *in vivo*, increasing expression of mesenchymal genes such as CD44, S100A10, ENG, VIM and ACTA2 (SMA), being the last one a typical marker of CAFs [4, 9].

CAF content and gene expression profiles related to mesenchymal features have been previously associated to agressiveness and poor prognosis in high-risk NBs [10, 11]. Nevertheless, the origin and function of these stromal cells remain unknown. In our work, we propose a major role for NC derived non-tumorigenic progenitor cells in the formation of such stromal compartment in aggressive NB. In line with this, *in silico* analysis of gene expression profiles from available tumor series confirmed the existence of a neural crest stem cell (NCSC) gene signature [13] associated to mesenchymal features and agressiveness in NB. K-means clustering of NB tumors based on the expression of NCSC genes resulted in three different groups with high (NCH), intermediate (NCI) and low (NCL) expression of this signature. Interestingly, NCH and NCL tumors showed increased probability of relapse compared to NCI tumors. Moreover, PAGE analysis demonstrated that NCH tumors were enriched in expression of genes clearly associated to a mesenchymal phenotype [48–50] . Gene ontology analysis revealed that genes overexpressed in NCH tumors were associated to biofunctions related to the stromal component of tumors, such as “extracellular matrix organization”, “immune response” or “angiogenesis”. In contrast, NCL tumor overexpressed genes are involved in functions related to cell proliferation and metabolism (data not shown). Our data also illustrate the correlation existing between SMA expression and poor prognosis in NB tumors. Moreover, we also demonstrate that high expression levels of NCSC gene signature correlate with high expression levels of ACTA2 (the gene encoding for SMA) in stage 4/M tumors. These results support the idea that elevated expression of mesenchymal genes observed in NCH tumors is mainly due to the presence of stromal cells being differentiated from NC progenitor cells.

Currently, one of the major challenges in NB research is to improve therapeutic strategies in aggressive tumors [3]. In our opinion, enrichment in NCSC gene signature and correlation to SMA expression could be useful to improve stratification of stage 4/M NBs. Tumors with high content of NC-derived stroma could be treated with novel therapeutic approaches targeting stroma-associated processes, such as immune response, cell migration or angiogenesis. Distinguishing these NC progenitor cell-containing tumors (NCH) from NCL tumors, in which aggressiveness more likely depends on tumorigenic neuroblast autonomous processes such as cell cycle, proliferation and metabolism, might also help devise better treatments for aggressive heterogeneous NB.

## MATERIALS AND METHODS

### Tumor dispersion and primary adherent cell cultures

The Hospital Virgen del Rocío Ethics Committee previously approved all protocols involving manipulation of human samples, and proper informed consents were obtained from subjects’ guardians. Patient-derived neuroblastoma fresh tumor samples (see Supplementary Table 1) were managed and obtained from *Hospital* *Universitario Virgen del Rocío/Instituto de Biomedicina de Sevilla Biobank (Andalusian Public Health System Biobank and* ISCIII-Red de Biobancos PT13/0010/0056). After discarding non-viable tissue, tumor samples were minced into small pieces (2–4 mm), washed with cold PBS (GIBCO) and enzymatically dissociated. Tumor fragments were incubated on dissociation solution (0.6 mg/mL Collagenase (Sigma-Aldrich), 0.05 mg/mL Elastase (Calbiochem, Merck Chemicals Ltd), 0.3 mg/mL Trypsin (Sigma-Aldrich) and 0.32 mg/mL DNAse I (Sigma-Aldrich), in HBSS buffer (GIBCO) in agitation at 37°C during 15–30 minutes. Tumor dispersion was completed mechanically by pipetting up and down. Then, cold fresh culture medium was added to stop the enzymatic reaction, and the resulting solution was filtered through a 70 μm cell strainer (BD Biosciences). When necessary, cell suspension was incubated 2–3 times in ACK buffer (155 mM NH_4_Cl, 2.96 mM NaHCO_3_ and 3.72 mM EDTA·4H_2_O) to eliminate erythrocytes. After that, viable cells were counted and seeded at the desire density in neural crest (NC) culture medium [14] containing DMEM:F-12 (GIBCO) with 15% fetal bovine serum (GIBCO), 1% N2 supplement (GIBCO), 1% B27 supplement (GIBCO), 100U penicillin:100mg streptomycin (GIBCO) per mL, 20 ng/ml recombinant human bFGF (R&D Systems), 20 ng/ml recombinant human IGF-1 (R&D Systems), and 20 ng/ml recombinant human EGF (R&D Systems).

### Sphere culture and differentiation protocol

Cells obtained after tumor dispersion were seeded in ultra low binding culture plates (Corning Inc.) at a density of 3 × 10^4^ – 5 × 10^4^ cells/ml in NC medium. To obtain spheres from established NB primary cell lines, after detaching cells from substrate using trypsin (Sigma-Aldrich), they were seeded in ultralow binding 6-well plates (Corning Inc.) at a clonal density of 5 × 10^3^ – 1 × 10^4^ cells/mL in NC medium. After 4–5 days in culture, spheres were passed to fresh NC medium. For all the experiments, spheres were grown for a minimum of 5 and a maximum of 10 days. For differentiation, after 4–5 days in culture, spheres were passed to fresh NC medium with or without serum and cultured for another 4–5 days in low binding culture plates. After that, spheres were passed to culture plates treated with 1–5 μg/cm^2^ of human fibronectin (Biomedical Technologies Inc.) and culture medium (with or without serum) was replaced. This step allowed spheres to attach to the substrate and grow in adherent conditions for another 4–5 days. Then, cells were fixed and analyzed by immunocytochemistry.

### Cell lines

IMR32, SK-N-SH and SK-N-DZ neuroblastoma cell lines were obtained from The American Type Culture Collection (*ATCC*) repository, and were cultured in Dulbecco's modified Eagle medium (DMEM; GIBCO), supplemented with 10% fetal bovine serum (FBS; GIBCO) and 100U penicillin:100mg streptomycin (GIBCO) per mL, at 37°C and 5% CO_2_. Neuroblastoma cell line SK-N-AS was obtained from the repository at Hospital Sant Joan de Déu (Barcelona, Spain). This line was grown in Roswell Park Memorial Institute (RPMI)-1640 medium supplemented with 10% FBS (Invitrogen), 2 mM L-glutamine, penicillin (100 U/mL) and streptomycin (100 μg/mL) (GIBCO), at 37°C and 5% CO_2_. Human fibroblasts (hFIB) cell line CCD 1112Sk was obtained from The American Type Culture Collection (*ATCC*) and cultured in Dulbecco's modified Eagle medium (DMEM; GIBCO), supplemented with 10% FBS (GIBCO) and 100U penicillin:100mg streptomycin (GIBCO) per mL, at 37°C and 5% CO_2_. Stable NB5t-EGFP or IMR32-RFP cell lines were obtained by transfection of lentiviral vectors expressing EGFP or RFP. For the co-cultures, IMR32-RFP and NB5t cells were mixed at 1:1 ratio and cultured onto adherent. The inhibitors AMD3100 (Sigma) and PAN-TGFβ (MAB1835; R&D Systems) were added at 20μM and 8mg/ml final concentrations, respectively.

### ET-1 treatment

Primary adherent cell lines cultured in NC medium were treated daily with 25 ng/mL ET-1 (Sigma-Aldrich) during 7 days and then fixed. Spheres were cultured in NC medium with low serum (1.5%) and treated daily with the same ET-1 concentration during 7 days. After that, we measured the number and diameter of the spheres and collected them for mRNA expression analysis. To determine the specificity of ET-1 effect in sphere cultures, endothelin receptors were blocked using Bosentan (gift from Actelion Pharmaceuticals). Bosentan was added daily at a final concentration of 10 mM, 1–2 hours before ET-1 treatment.

### Immunocytochemistry and immunohistochemistry

Immunocytochemical and immunohistochemical stainings were performed following standard procedures. For details, see Supplementary Materials and Methods.

### Flow cytometry

Flow cytometric analyses were performed in a BD LSRFortessa^TM^ cytometer (BD Bioscience), following standard procedures. For details, see Supplementary Materials and Methods.

### Total mRNA expression and gene ontology analysis

Total RNA was extracted from unfixed primary adherent cell lines and spheres using the RNeasy Mini Kit (Qiagen) following manufacturer's instructions. mRNA expression analysis was performed using GeneChip^®^ PrimeViewTM Human Gene Expression Arrays (Affymetrix). Expression changes induced by endothelin-1 treatment were determined by comparing the expression profiles of spheres obtained from 3 different primary cell cultures (NB5t, NB14t and NB27t) with or without ET-1 treatment. For each condition, we extracted and pooled the same quantity of total mRNA from 3 independent experiments. Results were analyzed using the Transcriptome Analysis Console (TAC) Software (Affymetrix), revealing changes in 660 probes (fold change > 1.5 and *p*-value < 0.05). To obtain biological significance from expression data, we performed a gene ontology analysis of differentially expressed genes using the Ingenuity Pathway Analysis (IPA) online toolkit (Ingenuity). Z-score is based on the direction of fold change values for genes in the input data set for which a correlation has been established in the Ingenuity Knowledge Database. Z-score > 2 predicts an increase in the activation state of cell function. All microarray rough analysis files are available from the GEO (http://www.ncbi.nlm.nih.gov/geo) database (accession number GSE78268).

### Quantitative real-time PCRs

Total RNA was extracted from unfixed cell cultures and tumor-derived tissues by RNeasy Mini Kit (Qiagen) following manufacturer's recommendations. Complementary DNA was generated using qScript™ cDNA SuperMix (Quanta Biosciences). PCRs were performed on ABI Prism 7700 Sequence Detector (Applied Biosystems) using Fast SYBR Green Master Mix (Applied Biosystem) and specific primers (listed in Supplementary Table 5) at a final concentration of 300 nM. In all samples, each gene was quantified at least in duplicate. Data were analyzed using the 2^-ΔΔCt^ method using GAPDH gene as housekeeping for normalization.

### Xenografts

All procedures involving mice were performed according to the animal care guidelines of the European Community Council (86/609/EEC) and were approved by the Animal Research Committee at the Instituto de Biomedicina de Sevilla/Hospital Universitario Virgen del Rocío (Sevilla, Spain). Xenografts were performed in six-week-old C.B-17 SCID mice (C.B-17/IcrHan^®^Hsd-*Prkdcscid*, Harlan Laboratories).

### Heterotopic xenografts

5×10^5^ neuroblastoma cells (IMR32 cell line) were injected subcutaneously in SCID mice, alone or with EGFP expressing primary cells from NB5t sample at a 1:1 ratio. For injection, cells were resuspended in a final volume of 100 μl: 50 μl of PBS (GIBCO) containing the cells were mixed with 50 μl of Matrigel (Corning Incorporated) and injected subcutaneously using a 29-gauge needle. After 45–60 days, animals were sacrificed and tumors were removed and processed as indicated above.

### Orthotopic xenografts

Female mice were anaesthetized using 100 mg/Kg ketamine and 5 mg/Kg diazepam injected intra-peritoneally. Orthotopic implantation was performed through ventrolateral incision. A total of 1×10^6^ NB cells in 10 μl of PBS (GIBCO) were injected in the left adrenal gland using a 33-gauge needle connected to a 10 μl 701 RN SYR syringe (Hamilton). Abdominal wall and skin were closed with absorbable suture (VICRYL 4/0, Ethicon) and silk suture (SILKAM 4/0, Braun surgical) respectively. After surgery, the animals were treated with 1mg/Kg flumazenil injected intramuscularly to revert the effect of diazepam, and were analgesized with 0.3 mg/Kg meloxicam injected subcutaneously. Xenografted animals were usually sacrificed after one month. Exceptionally, and only in those cases in which we injected tumor-derived primary cells that finally were revealed as non-tumorigenic, we waited up to six months before seeking for tumor formation.

### Determination of haploid *MYCN* copy number by quantitative real-time PCR (qPCR)

Genomic DNA from adherent primary cell cultures and commercial NB cell lines was isolated using the GenElute^TM^ Mammalian Genomic DNA miniprep kit (Sigma-Aldrich) following manufacturer's instructions. Analysis of *MYCN* amplification status was performed by means of qPCR using TaqMan detection chemistry as previously described [58]. Briefly, *MYCN* and two reference genes, *BCMA* and *SDC4,* were simultaneously amplified in separate tubes for each tumor sample. PCR reactions were performed on an ABI Prism Sequence Detection System 7500 SDS (Applied Biosystems). Duplicate amplification mixtures for each sample (10 μL) were prepared for *MYCN*, *BCMA* and *SDC4* quantification, and contained genomic DNA (10 ng), Taqman mix, 300 nM of each primer, and 200 nM of the probe. Haploid *MYCN* copy number was calculated according to 2^-ΔΔCt^ method. Genomic DNA from neuroblastoma cell line LA-N-1 was used as positive control for *MYCN* amplification. Genomic DNA isolated from blood of 3 healthy donors was pooled and used as calibrator sample.

### Multiplex ligation-dependent probe amplification (MLPA)

Genomic DNA from different samples was isolated using the GenElute^TM^ Mammalian Genomic DNA miniprep kit (Sigma-Aldrich) following manufacturer's instructions. Quantification of losses, gains and amplifications were determined by multiple ligation- dependent probe amplification (MLPA) according to the methods previously reported [59]. Each genomic DNA sample was amplified and analyzed using the neuroblastoma-specific MLPA kit SALSA P251-B1 (MRC Holland), which contains 34 probes for chromosomes 1, 3, and 11, as per manufacturer's instructions. Genomic DNA from peripheral blood of 3 healthy donors was pooled and used as reference. Also, DNA from neuroblastoma cell line SK-N-AS was used as positive control.

### Bioinformatic analysis of neuroblastoma tumor series

All bioinformatic analyses were performed *in silico* using expression data from tumor series stored in the R2 bioinformatic platform, available from the public website http://R2.amc.nl. Details about these analyses are given in Supplementary Materials and Methods.

### Statistics

Bar charts represent mean ± S.E.M. Box and whisker plots represent the median (line), 25% and 75% percentiles (box), and minimum and maximum values (whiskers). In Figures 1–3 and Supplementary Figure 3, (n) represents technical replicates, while biological replicates are shown as individual symbols. In Figures 4–6 (n) represents biological replicates. Statistical and correlation tests are indicated in each case. All statistics were performed using Prism 6.0 (GraphPad Software), except Kaplan-Meier curves, PAGE analysis and Gene Ontology studies, in which statistical significance was provided automatically by the on-line software R2 analysis and visualization platform (http://R2.amc.nl), or by the Ingenuity Pathway Analysis on-line toolkit. For data shown in Figure 3H, we previously eliminated outliers by applying the Grubbs’ test (alpha = 0.05).

## SUPPLEMENTARY MATERIALS FIGURES AND TABLES




