# Supplementary material for: Neural crest derived progenitor cells contribute to tumor stroma and aggressiveness in stage 4/M neuroblastoma
Source: Oncotarget. 2017 Sep 21;8(52):89775–92. doi: 10.18632/oncotarget.21128 (PMC5685708; doi:10.18632/oncotarget.21128)
Supplement: Supplementary file 1 [file oncotarget-08-89775-s001.pdf]

Neural crest derived progenitor cells contribute to tumor stroma and aggressiveness in stage 4/M neuroblastoma

SUPPLEMENTARY MATERIALS

MATERIALS AND METHODS

Immunocytochemistry

Cells were fixed with 4% PFA (Sigma-Aldrich) and then pre-blocked for 1 h at room temperature in blocking solution:PBS (GIBCO) containing 10% goat serum (Sigma-Aldrich), 0.1% BSA (Sigma-Aldrich), and 0.3% Triton X-100 (Sigma-Aldrich). Primary antibodies (See Supplementary Table 4) diluted in blocking solution were incubated overnight at 4°C, followed by fluorochrome-conjugated secondary antibodies for 2 hours at room temperature. Finally, slides were counter stained with 2.5 mg/ml DAPI (Sigma-Aldrich). Quantification of cell number (nuclei) and expression of Bmi1, Msi1, Oct4, Nestin, PHH3 and Ki67 was performed using Cell Profiler software [1] and custom-made pipelines. Quantification of cells positive for Nestin and/or GFAP in Figure 2 and Supplementary Figure 2 was performed using ImageJ. Asymmetric distribution of GFAP and Nestin shown in Supplementary Figure 2 was measured using ImageJ. We manually drown a 30µm line along cell division axis, and measured fluorescence intensity (arbitrary units) on each pixel of the line, for blue (DAPI), green (Nestin) and red (GFAP) channels.

Flow cytometry

Cells from tumor-derived primary cultures and NB cell lines were detached using trypsin and resuspended in staining buffer containing (for 50 ml): 44 ml L15 medium (GIBCO), 0.5 ml penicillin/streptomycin (GIBCO), 0.5 ml 1M HEPES buffer (GIBCO), 0.1g BSA (Sigma), and 5 ml distilled and deionized water (Sigma-Aldrich). Primary antibodies (indicated in Supplementary Table 4) were incubated at 4ºC for 1 hour and then washed. When needed, cells were resuspended and incubated at 4ºC for 1 hour in staining medium containing appropriate secondary antibodies conjugated to fluorescein isothiocyanate (FITC) or phycoerythrin (PE) (indicated in Supplementary Table 4). After washing off unbound antibodies, cells were resuspended in staining medium containing 2 mg/ml 7-AAD (Molecular Probes). Dead cells (positive for 7-AAD staining) were eliminated from analyses. Analysis of cells positive for each antibody was performed in a BD LSRFortessaTM cytometer (BD Bioscience).

Xenografted tumors were processed as previously described for patient-derived tumor samples. Dispersed cells were resuspended in staining solution, and EGFP endogenous fluorescence was measured in a BD FACSCantoIITM cytometer (BD Biosciences).

Immunohistochemistry

35 tumor samples from 27 patients diagnosed as neuroblastoma at the Department of Pediatric Oncology, Virgen del Rocío University Hospital (Sevilla, Spain) between July 2011 and October 2014 (See Supplementary Table 3) were formalin-fixed and embedded in paraffin. Tumor samples obtained from xenografted mice were fixed in 4% PFA (Sigma-Aldrich) in PBS (GIBCO) at 4 °C overnight, rinsed in 70% ethanol and processed for paraffin embedding in a Leica ASP200 S tissue processor (Leica). From each sample, pathologists reviewed hematoxylin/eosin-stained sections and two different representative regions (1 mm diameter cores) were selected for tissue microarray (TMA) construction. TMA sections (3 µm) were de-paraffinized in xylene, rehydrated and rinsed with PBS (GIBCO). Antigen retrieval was performed by pre-treating sections with citrate buffer 10 mM (pH 6.0) and thermally processing them with two cycles in a 2100 Antigen Retriever (Aptum Biologics). After that, sections were washed in distilled water. For 3,3-diaminobenzidine (DAB) staining, endogenous peroxidase was blocked using 0.3% hydrogen peroxide (Sigma-Aldrich) in distilled water for 10 minutes at room temperature, washed with distilled water and finally rinsed with PBS (GIBCO). Sections were permeabilized and blocked in 0.2% Triton X-100 (Sigma-Aldrich), 3% normal goat serum (GIBCO) and 1% BSA (Sigma-Aldrich) in PBS (GIBCO) for 45 minutes at room temperature. Primary antibodies (See Supplementary Table 4) were incubated in the same blocking solution overnight at 4ºC. After washing to eliminate unbound antibodies, sections were incubated with the appropriate biotinylated secondary antibodies (See Supplementary Table 4), diluted in blocking solution, during 45 minutes at room temperature. After washing, sections were incubated with the avidin-biotin-peroxidase (ABC) complex Vectastain Elite ABC kit (Vector Labs) for 30 minutes at room temperature. Staining was revealed using 3,3′-diaminobenzidine (DAB)-EnVision FLEX kit (DAKO). Finally, TMA sections were washed, counterstained with hematoxylin (Bio Optica), dehydrated, and mounted with DPX mountant (Sigma). For immunofluorescence, after antigen retrieval, sections were permeabilized and blocked in 0.2% Triton X-100 (Sigma-Aldrich), 3% normal goat serum (GIBCO) and 1% BSA (Sigma-Aldrich) in PBS (GIBCO) for 45 minutes at

room temperature. Primary antibodies (See Supplementary Table 4) were incubated in the same blocking solution overnight at 4ºC. After washing to eliminate unbound antibodies, sections were incubated with the appropriate fluorochrome-conjugated secondary antibody (See Supplementary Table 4) during 1 hour at room temperature, and then mounted with Fluoroshield mounting medium with DAPI (Sigma-Aldrich). Quantification of DAB staining was performed using ImageJ.

Bioinformatic analysis of neuroblastoma tumor series

All bioinformatic analyses were performed in-silico using expression data from tumor series stored in the R2 bioinformatic platform, available from the public website http://R2.amc.nl. We used data from stage 4 high-risk neuroblastoma tumors belonging to two different tumor series. Analysis shown in Figures 5 and 6 was done using the expression data from stage 4 neuroblastoma tumors (n = 214) included in a tumor series containing expression data from 649 tumor samples (GEO ID: GSE45547) [2]. To confirm the results, we used a second tumor series containing 102 stage 4 non-MYCN amplified tumors (GEO ID: GSE3446) [3] (see Figure 6). All the analyses were performed using default parameters provided by R2 platform. Tumors were clustered using k-means analysis attending to the expression level of genes up-regulated in neural crest stem cells (NCSCs), as defined by Lee et al. (Broad Institute, Gene Set: LEE_NEURAL_CREST_STEM_CELL_UP) [4]. The analysis was repeated 100 times to select the clustering with the lowest distance to the mean. This process was repeated 10 times, allowing evaluation of consistency of the outcomes. Tumors were clustered in three different groups defined by the expression of the signature (High, Intermediate and Low) and the clustering was repeated using genes within the NCSC Gene Set (n = 87) that were differentially expressed between NCSC High and NCSC Low tumors. This classification was used to perform subsequent analyses. Single gene expression quantification was performed in each individual group after 2log transformation of expression data. Transcriptome-wide expression profile analysis was performed by principal component analysis (PCA). Kaplan-Meier survival curves were calculated using tumor samples with available survival data. Tumors samples were grouped attending to the expression of the NCSC Gene Set (High, Intermediate and Low) or automatically separated in two different groups attending to the expression level of ACTA2. The p-value for survival curves was calculated as previously described [5]. For gene set enrichment analysis, besides NCSC gene set defined by Lee et al. [4], we used three different gene sets associated to the mesenchymal phenotype of cancer: genes up-regulated in a mesenchymal subtype of glioblastoma (Broad Institute, Gene Set: VERHAAK_GLIOBLASTOMA_MESENCHYMAL) [6], genes expressed in stromal stem cells (Broad Institute, Gene Set: BOQUEST_STEM_CELL_UP) [7] and genes involved in mesenchymal transition in cancer (Broad Institute, Gene set: ANASTASSIOU_CANCER_MESENCHYMAL_TRANSITION_SIGNATURE) [8]. The expression values of all genes from a gene set were transformed into Z-scores and subsequently averaged over all the members of the gene set. This resulted in a single value for a gene set, for every sample in a dataset. This procedure was then repeated for every single gene set selected for the analysis, hierarchically clustered and plotted in a heatmap. For every single gene set, enrichment analysis comparing NCSC High vs NCSC Intermediate+Low tumors was done using the PAGE (Parametric Analysis of Gene set Enrichment) algorithm [9]. For correlations, we obtained individual tumor values for gene set average Z-scores or log2 transformed gene expression. Each pair of analyzed values was plotted and Pearson´s correlation coefficient was calculated. For gene ontology (GO) analysis, we first calculated the genes that were differentially expressed between NCSC High and NCSC Intermediate+Low tumors. This analysis uncovered the existence of 8025 differentially expressed genes between both groups (ANOVA, p < 0,01) that were analyzed using the GO analysis tool available in the R2 platform.

REFERENCES:

1. Carpenter AE, Jones TR, Lamprecht MR, Clarke C, Kang IH, Friman O, Guertin DA, Chang JH, Lindquist RA, Moffat J, Golland P, Sabatini DM. CellProfiler: image analysis software for identifying and quantifying cell phenotypes. Genome biology. 2006; 7:R100.
2. Kocak H, Ackermann S, Hero B, Kahlert Y, Oberthuer A, Juraeva D, Roels F, Theissen J, Westermann F, Deubzer H, Ehemann V, Brors B, Odenthal M, et al. Hox-C9 activates the intrinsic pathway of apoptosis and is associated with spontaneous regression in neuroblastoma. Cell death & disease. 2013; 4:e586.
3. Asgharzadeh S, Pique-Regi R, Sposto R, Wang H, Yang Y, Shimada H, Matthay K, Buckley J, Ortega A, Seeger RC. Prognostic significance of gene expression profiles of metastatic neuroblastomas lacking MYCN gene amplification. Journal of the National Cancer Institute. 2006; 98:1193–1203.
4. Lee G, Kim H, Elkabetz Y, Al Shamy G, Panagiotakos G, Barberi T, Tabar V, Studer L. Isolation and directed differentiation of neural crest stem cells derived from human embryonic stem cells. Nature biotechnology. 2007; 25:1468–1475.
5. Bewick V, Cheek L, Ball J. Statistics review 12: survival analysis. Critical care. 2004; 8:389–394.
6. Verhaak RG, Hoadley KA, Purdom E, Wang V, Qi Y, Wilkerson MD, Miller CR, Ding L, Golub T, Mesirov JP, Alexe G, Lawrence M, O'Kelly M, et al. Integrated genomic analysis identifies clinically relevant subtypes of

glioblastoma characterized by abnormalities in PDGFRA, IDH1, EGFR, and NF1. Cancer cell. 2010; 17:98–110.

7. Boquest AC, Shahdadfar A, Fronsdal K, Sigurjonsson O, Tunheim SH, Collas P, Brinchmann JE. Isolation and transcription profiling of purified uncultured human stromal stem cells: alteration of gene expression after in vitro cell culture. Molecular biology of the cell. 2005; 16:1131–1141.

8. Anastassiou D, Rumjantseva V, Cheng W, Huang J, Canoll PD, Yamashiro DJ, Kandel JJ. Human cancer cells express Slug-based epithelial-mesenchymal transition gene expression signature obtained in vivo. BMC cancer. 2011; 11:529.

9. Kim SY, Volsky DJ. PAGE: parametric analysis of gene set enrichment. BMC bioinformatics. 2005; 6:144.

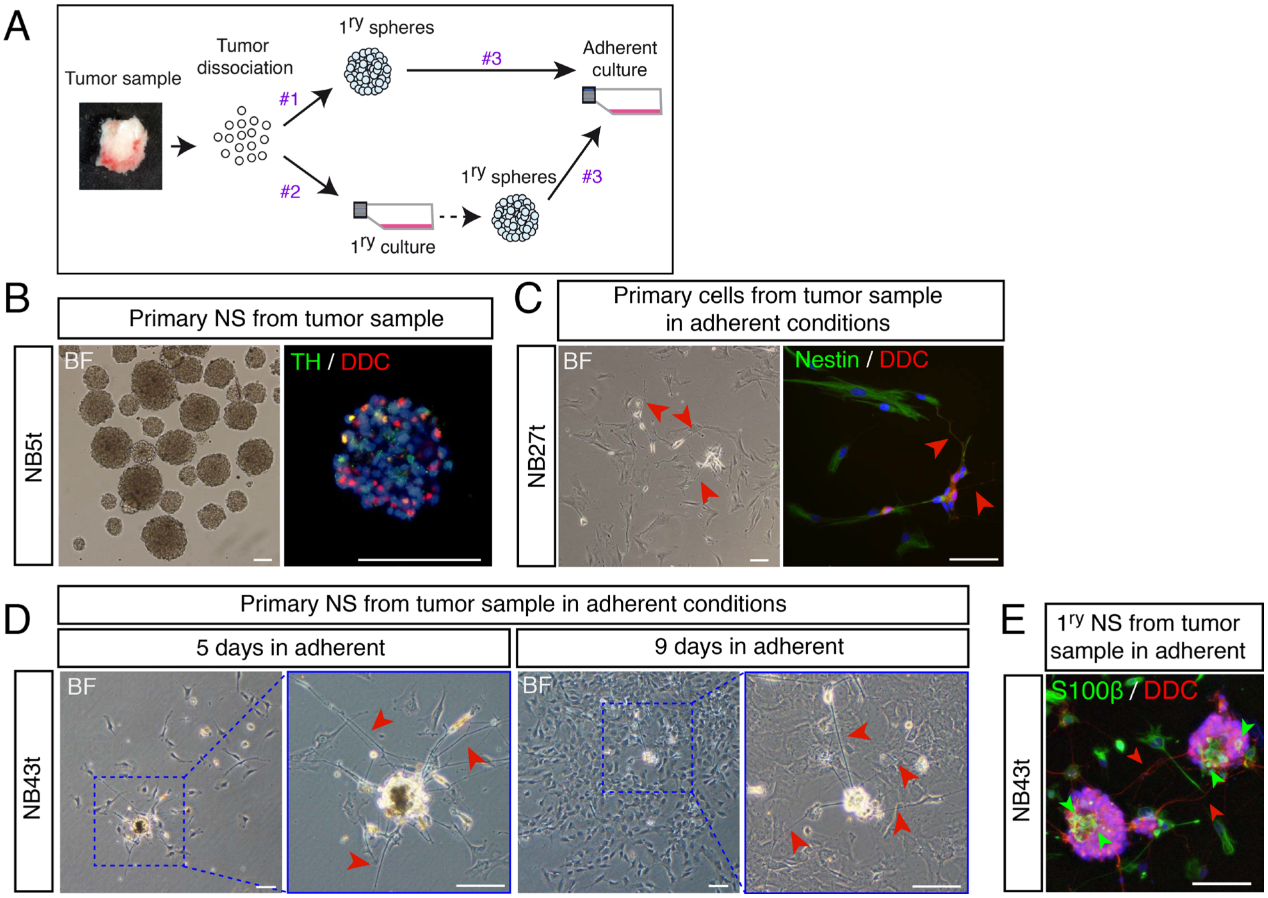

Supplementary Figure 1: Presence of neuroblasts in primary cell cultures isolated from NB tumor biopsies. After tumor dissociation, neuroblasts grew in suspension as spheres but tended to differentiate when attached to the substrate, disappearing from subsequent passages. (**A**) Schematic view of tumor cell isolation protocol. Dispersed tumor cells were cultured in low binding conditions for the formation of neurospheres, either directly (#1) or through adherent conditions (#2). In all cases, spheres were passed back to adherent cultures (#3) for the selection of stromal cells. (**B**) Representative bright field image of primary spheres grown in low binding conditions (option #1 in (A)) (left panel). Immunohistochemistry (right panel) revealed the neuroblastic phenotype of primary spheres. A representative sphere section is shown labeled for nuclei (blue), tyrosine hydroxylase (TH; green) and dopamine decarboxylase (DDC; red). (**C**) Representative bright field image of primary adherent culture (left panel) obtained directly from tumor dissociated cells (option #2 in (A)). Together with stromal-like cells, we initially observed cells with neuroblastic phenotype and with emission of typical neurites characteristic of neuronal differentiation (red arrowheads). The presence of neuroblasts was confirmed by immunofluorescence (right panel). Image show labeling for nuclei (blue), nestin (green) and dopamine decarboxylase (red). Dopamine decarboxylase positive neurites are highlighted (red arrowheads). (**D**) Final adherent primary cultures were obtained by culturing primary spheres in adherent conditions (step #3 in (A)). Images show representative bright fields of cultures generated after passing primary spheres to adherent substrate. After attaching to the substrate, neuroblastic cells within the spheres tended to differentiate and emit neurites (red arrowheads). Interestingly, images show how after 5 days in adherent conditions we observed stromal-like cells surrounding spheres (left panels). These cells divide and generate primary cultures formed mainly by stromal-like cells. Neuroblasts within the spheres clearly tended to differentiate (neurites; red arrowheads) and disappeared in subsequent passages in favour of highly-proliferative stromal-like cells. (**E**) Representative confocal image of adherent spheres showed in (D). The image shows labeling for nuclei (blue), S100b (green), and dopamine decarboxylase (red). DDC positive neurites are highlighted (red arrowheads). Stromal cells are positive for S100b. Interestingly, some S100b positive cells are observed within spheres together with DDC positive cells (green arrowheads). These results suggest that primary neurospheres obtained from NB tumors contain both neuroblast-like and stromal-like cells, and that stromal cells take over the cultures in subsequent adherent passages. Scale bars: 100 µm.

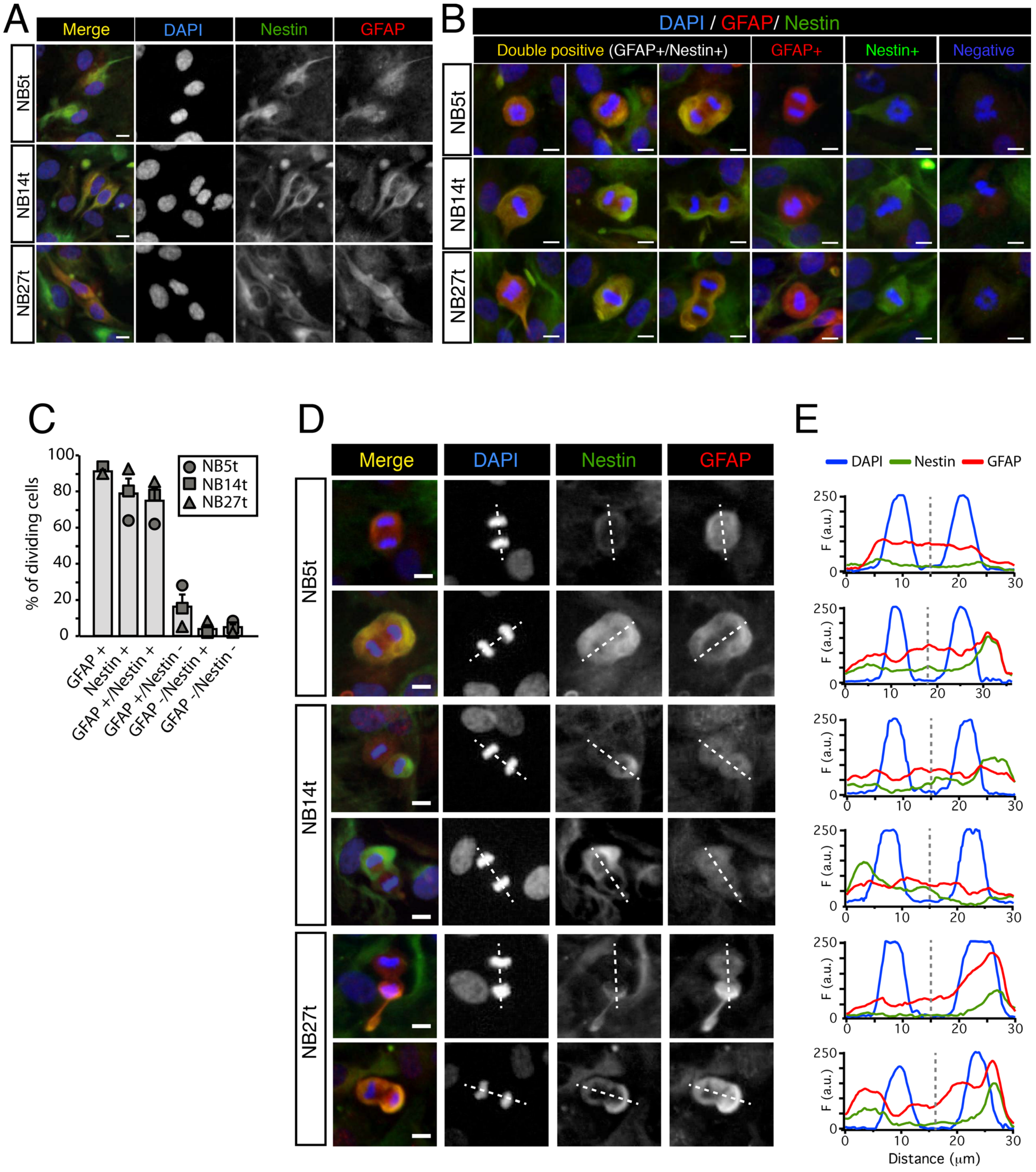

Supplementary Figure 2: Phenotypical characterization of proliferating cells in NB derived adherent cultures. Immunofluorescent images of adherent cells obtained from three different NB tumor biopsies, showing expression of typical neural stem cell markers. (**A**) Representative images of cells double positive for GFAP (red) and Nestin (green). Nuclei are labeled in blue. (**B**) Representative examples of dividing cells (DAPI staining clearly reveals chromosome condensation) immunolabeled against GFAP (red) and Nestin (green). Different existing phenotypes regarding expression of GFAP and Nestin are shown. (**C**) Quantification of dividing cells attending to the expression of GFAP and/or Nestin. 74.8 ± 6.7% of all dividing cells in adherent conditions were double positive for GFAP and Nestin, indicating a mayor role of these neural stem cells in proliferation and growth of primary cultures. (**D**) Immunofluorescence images of adherent cells obtained from three different NB tumor biopsies showing expression of the typical neural stem cell markers GFAP and Nestin. In some cases, the distribution of GFAP and Nestin labeling between the daughter cells highlighted the existence of asymmetric cell divisions, an evolutionary conserved division mode used by stem and progenitor cells. (**E**) Measurement of GFAP and Nestin labeling intensity. We drew a line coinciding with the division axis (dotted lines in (D)), and measured the fluorescence intensity (arbitrary units) in blue (nuclei), green (Nestin) and red (GFAP) channels. Scale bars = 10 μm.

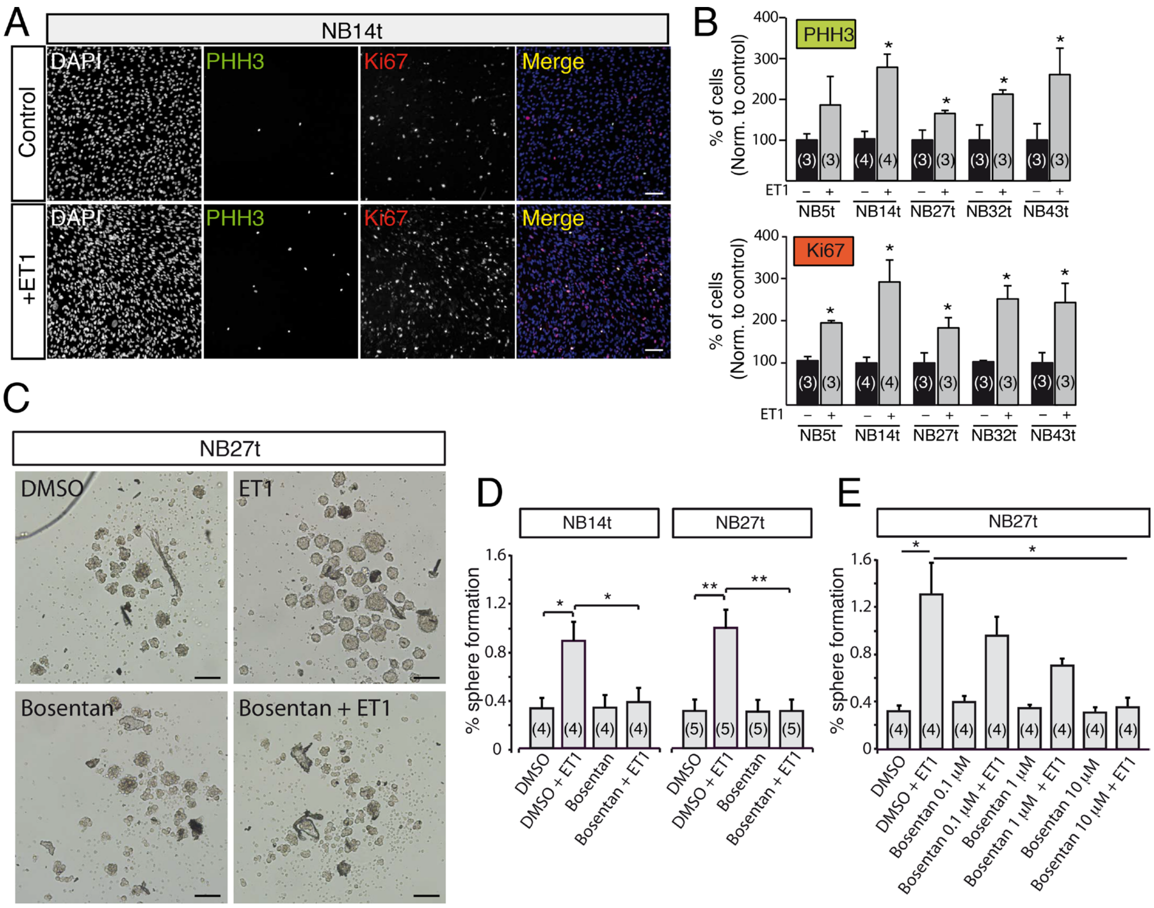

Supplementary Figure 3: NB derived neural crest progenitors are sensitive to ET-1 treatment. (**A**) Representative immunofluorescence images of NB derived adherent cells with or without ET-1 treatment. Proliferative cells were detected using antibodies against phospho-histone H3 (PHH3; green) and Ki67 (red). Nuclei are shown in blue. (**B**) Quantification of the percentage of cells positive for PHH3 and Ki67 in five different NB-derived adherent cell cultures. Values are normalized to the number of proliferative cells in control conditions for every single cell line analyzed. (**C**) Representative bright field images showing sphere cultures from sample NB27t treated with ET-1 and with ET-1 plus bosentan (a blocker of endothelin receptors). (**D**) Quantification of the increase in the % of sphere formation mediated by ET-1 and in the presence of ET receptor blocker bosentan. (**E**) Dose-response curve of bosentan effect on blocking the increase in the % of sphere forming cells induced by ET-1. *$p < 0.05$, **$p < 0.01$, Student's t-test. Scale bars = 100 μm.

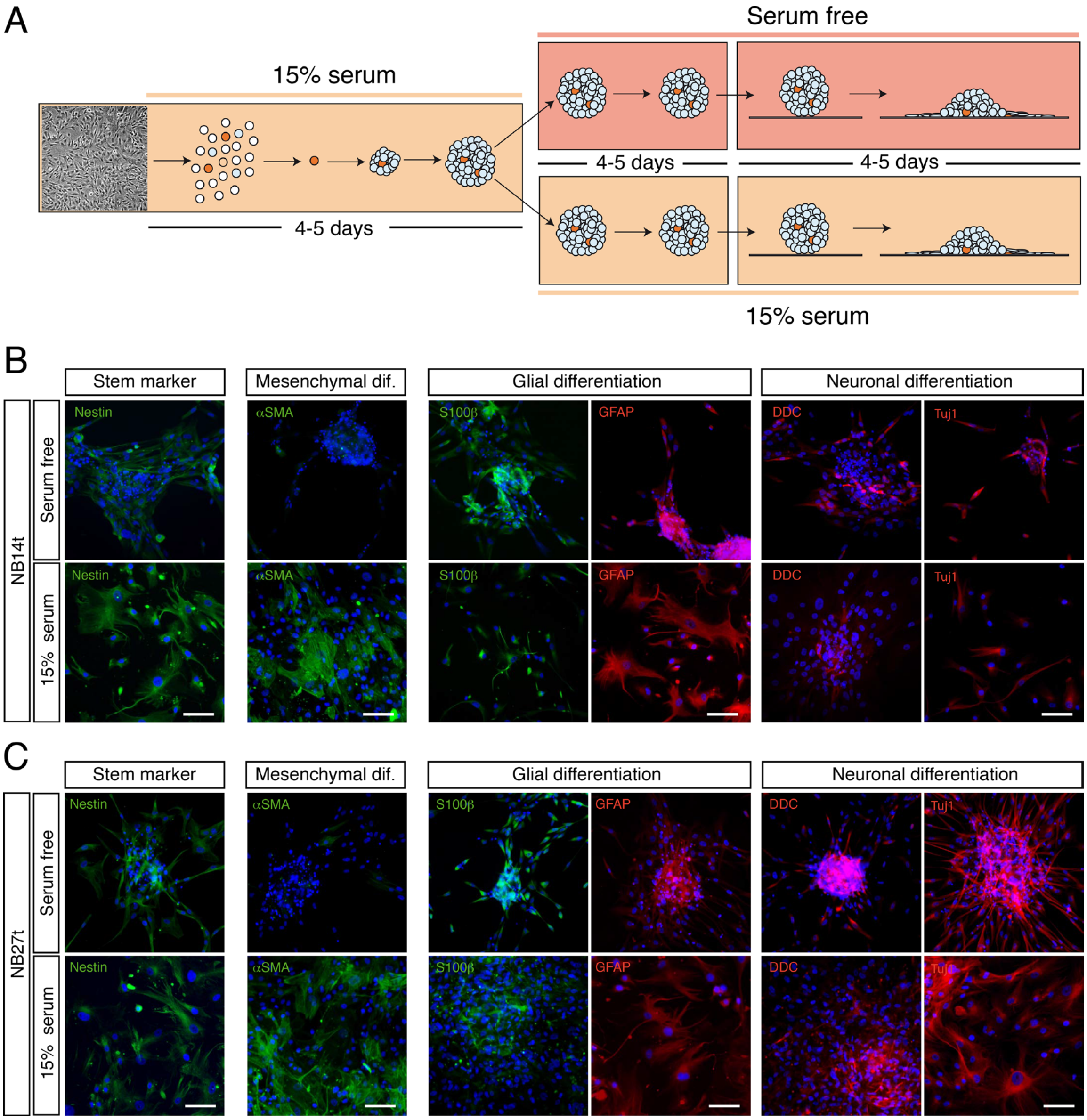

Supplementary Figure 4: NB derived neural crest progenitors can differentiate into both neural and mesectodermal lineages. (**A**) Schematic view of differentiation protocol. (**B**, **C**) Immunofluorescent staining of NB-derived spheres after 4-5 days of differentiation in adherent conditions with or without 15% serum. Cells were labeled with antibodies against Nestin (stem cell marker), SMA (mesenchymal differentiation marker), S100b and GFAP (glial differentiation markers), and DDC and Tuj1 (neuronal differentiation markers). Scale bars = 100 µm.

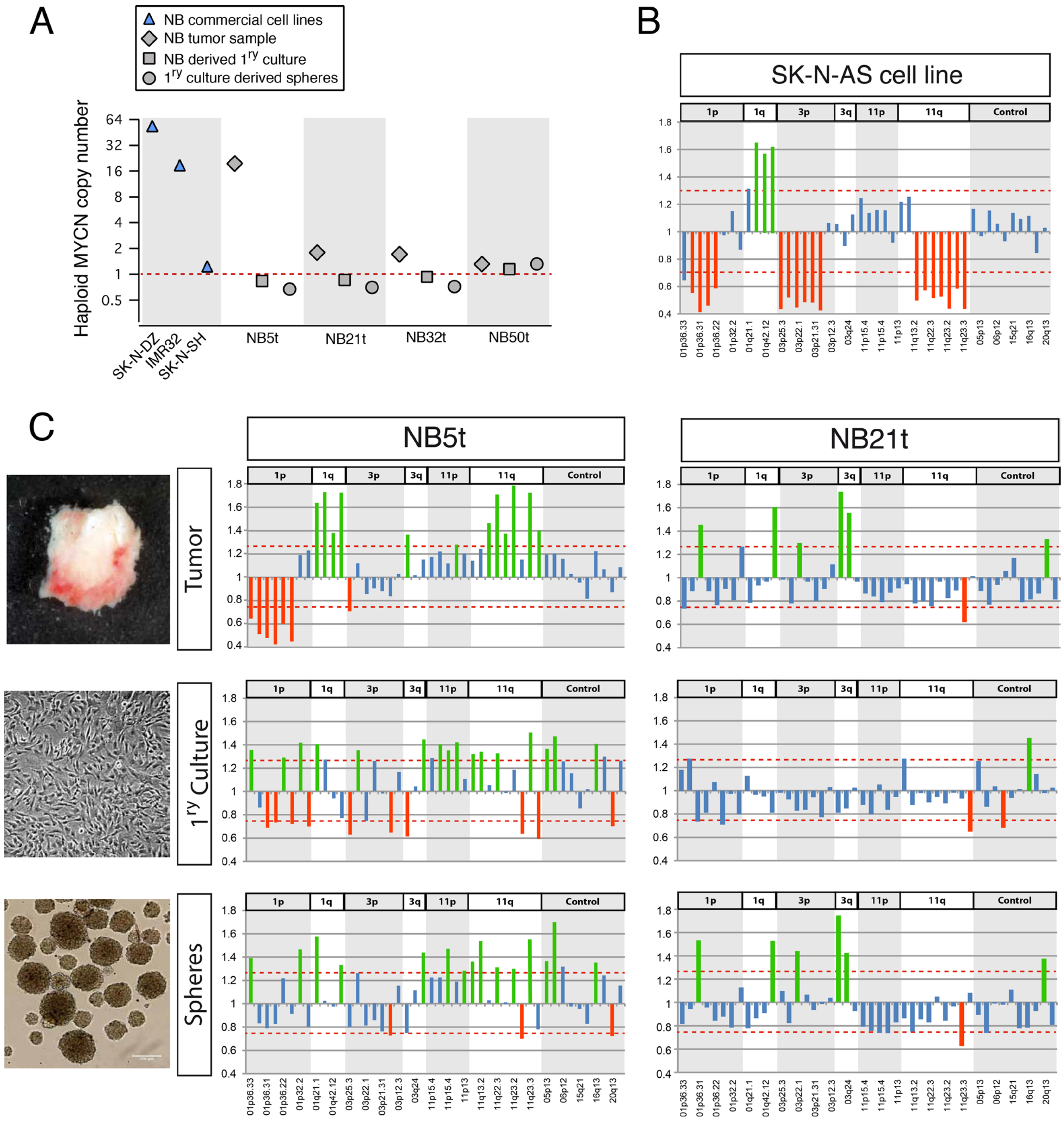

Supplementary Figure 5: Genomic analysis of NB derived primary cells. (**A**) Quantification of MYCN haploid copy number by qPCR. Tumor samples, primary cells from tumor biopsies, and primary cells-derived spheres from four different NB samples were analyzed. As controls, we used cells from SK-N-DZ, IMR32 (both MYCN amplified) and SK-N-SH (MYCN non amplified) commercial NB cell lines. (**B**, **C**) Quantification of chromosomic losses and gains by multiple ligation-dependent probe amplification (MLPA) analysis. (B) SK-N-AS cell line was used as control. (C) MLPA analysis of tumor samples, primary cell cultures from tumor biopsies and primary cell-derived spheres from two NB tumor samples (NB5t and NB21t). The results demonstrated the absence in primary cells and spheres of critical genomic alterations observed in tumor samples.

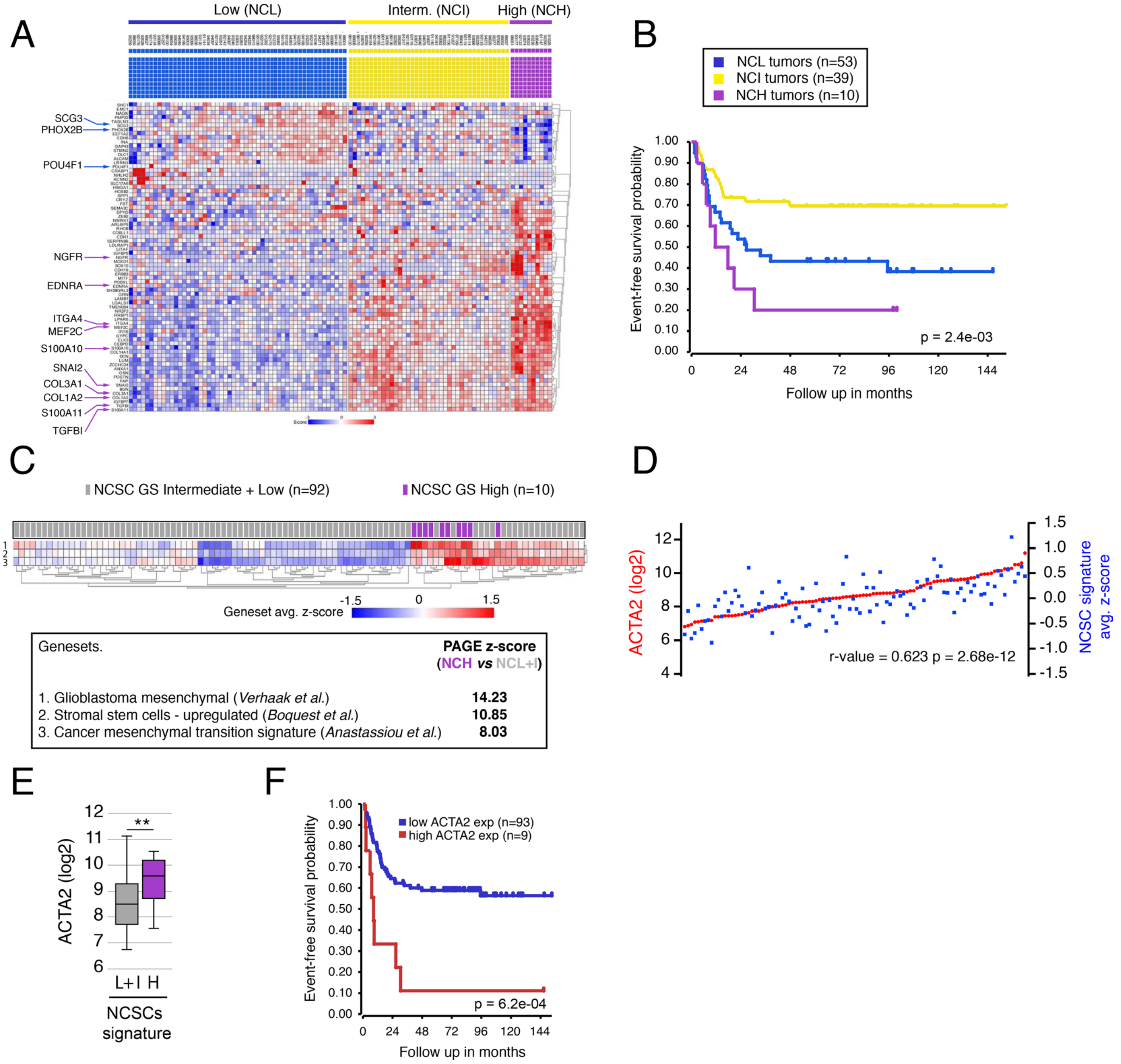

Supplementary Figure 6: Neural crest stem cell (NCSC) gene signature and ACTA2 expression in an additional stage 4/M NB tumor series. (**A**) K-means clustering using genes up-regulated in NCSCs (described in Lee et al. [4] (Suppl. Ref.)) segregate stage 4/M NB tumors into three different groups depending on the high, intermediate or low expression levels of the signature (NCH, NCI and NCL tumors respectively). (**B**) Kaplan-Meier curves showing the event-free survival probability of NCH, NCI and NCL tumors. NCH and NCL tumors present a high probability of relapse compared to NCI neuroblastomas. (**C**) Parametric analysis of geneset enrichment (PAGE) revealed enrichment in NCH tumors of genes previously described as characteristic of mesenchymal cells. Enrichment z-score for three different genesets is shown: genes typical of the mesenchymal subtype in glioblastoma (geneset #1, described in Verhaak et al. [6] (Suppl. Ref.)), genes up-regulated in stromal stem cells (geneset #2, described in Boquest et al. [7] (Suppl. Ref.)) and genes typical of mesenchymal transition in cancer (geneset #3, described in Anastassiou et al. [8] (Suppl. Ref.)). Geneset map (heatmap in the upper panel) of average z-scores for all genes in every single geneset calculated for each individual sample. (**D**) Correlation between ACTA2 expression and NCSC signature average z-scores in individual tumors over the whole sample of tumors analyzed. (**E**) Box-whiskers plots showing the expression of ACTA2 in tumors classified as NCH or NCL+I, depending on their NCSC signature enrichment. Tumors enriched in expression of NCSC genes (NCH) show higher expression levels of ACTA2. ** $p < 0.01$, Student's t-test. (**F**) High ACTA2 expression levels are associated to lower event-free survival probability.

Supplementary Table 1: Tumor samples used for primary cultures

			Clinical Data						
**Sample**	**Stage**	**Localization of primary tumor**	**Age at diagnosis (months)**	**Metastasis**	**Relapse/ Progression**	**Pre-post treatment**	**Mortality**	**NMYC Amp**	**Deletion 1p36**
NB5t	4/M	Retroperitoneal (RP)	32	YES	YES	POST (RELAPSE)	NO	YES	YES
NB13t	4/M	Thoracic/Paraspinal	26	YES	NO	POST	YES	NO	-
NB14t<br>NB21t	4/M	Suprarenal /Left	11	YES	NO	PRE<br>POST	NO	NO	YES
NB27t	4/M	Cervico-thoracic (CT)	60	YES	YES	POST (RELAPSE)	YES	YES	YES
NB28t<br>NB32t	4/M	Retroperitoneal (RP)	10	YES	NO	PRE<br>POST	NO	YES	YES
NB43t	3, L2	Retroperitoneal (RP)	83	YES	YES	PRE	YES	NO	NO
NB50t	4/M	Cervical	48	YES	NO	POST	NO	NO	NO
NB58t	4/M	Suprarenal /Left	48	YES	NO	PRE	NO	NO	NO

Supplementary Table 2: Primary cells used for xenografts.

1ry Cell lines	Xenograft type	No. of mice injected with cells from		No of tumors	Efficiency
		Adherent cell culture	Spheres		
NB5t	Orthotopic	-	3	0	0%
	Heterotopic	6	6	0	0%
NB14t	Orthotopic	-	-	-	0%
	Heterotopic	2	-	0	0%
NB21t	Orthotopic	-	4	0	0%
	Heterotopic	-	-	-	0%
NB27t	Orthotopic	-	-	-	0%
	Heterotopic	4	4	0	0%
NB28t	Orthotopic	-	-	0	0%
	Heterotopic	4	4	0	0%
NB58t	Orthotopic	-	3	0	0%
	Heterotopic	4	-	0	0%
Total	Orthotopic	-	10	0	0%
	Heterotopic	20	14	0	0%
Cell line					
IMR32	Orthotopic	3	-	3	100%
	Heterotopic	8	4	10	83.3%

Supplementary Table 3: Clinical information for samples included in TMAs. See Supplementary_ Table_3

Supplementary Table 4: Antibodies used for immunohistochemistry (IHC), immunocytochemistry (ICC) and flow cytometry (FC)

Protein/Epitope	Primary antibodies	Application (dilution)	Company (#Ref)
Undetermined. Specific for human fibroblasts.	Anti-Fibroblasts (Clone TE-7)	ICC (1/500)	Millipore (CBL271)
aSMA	Anti-aSMA (clone 1A4)	ICC (1/1000), IHC (1/10000)	Sigma (A2547)
NCAM1 (CD56)	Anti-NCAM (RNL-1)	FC (1/100)	Abcam (ab9018)
HNK1 (CD57)	Anti-Human CD57	FC (1/200)	BD Pharmingen (559048)
NGFR(p75, CD271)	Anti-Nerve Growth Factor Receptor (Clone MLR2)	FC (1/100)	Millipore (MAB5592)
NGFR(p75, CD271)	Anti-NGF receptor p75	ICC (1/500)	Millipore (AB1554)
Thy1 (CD90)	CD90 (Clone 5E10)	ICC (1/500), FC (1/100)	BD Pharmingen (555593)
MME, Neprilysin (CD10)	Human Neprilysin/CD10 antibody	FC (1/50)	R&D Systems (AF1182)
MSCA1	Anti-Human MSCA1-PE Conjugated	FC (1/11)	Miltenyi Biotec (130-099-198)
CD44	Anti-Human CD44	ICC, FC (1/200)	BD Pharmingen (555476)
ENG (CD105)	Anti-Human CD105-APC Conjugated	FC (1/11)	Miltenyi Biotec (130-099-125)
hNestin	Anti-Human Nestin	ICC (1/500), IHC (1/500)	R&D Systems (MAB1259)
GFAP	Anti-GFAP (Polyclonal Rb)	ICC (1/500)	Dako (Z0334)
		IHC (1/500)	Millipore (AB5804)
S100b	Anti-S100 (β subunit)	ICC (1/500)	Sigma (S2532)
b3-tubulin	Anti-b3 tubulin (Tuj1)	ICC (1/1000)	Abcam (ab18207)
DDC	Anti-DOPA Decarboxilase	ICC (1/500)	Millipore (AB1569)
Bmi1	Anti-Bmi1	ICC (1/500)	Abcam (ab38295)
Msi1	Anti.Musashi/Msi1	ICC (1/500)	Abcam (ab21628)
Oct4	Anti-Oct4	ICC (1/500)	Abcam (ab18976)
Histone-H3 (phosphorylated)	Anti-Phospho-Histone H3 (Ser28)	ICC (1/500)	Cell Signaling (9713)
Ki67	Ki67 (Clone SP6)	ICC (1/500)	Thermo (RM-9106-S0)
Green Fluorescent Protein	Anti-GFP	IHC (1/5000)	Synaptic Systems (132 003)
TH	Anti-Tyrosine Hidroxilase	IHC (1/1000)	Sigma (T1299)
EDNRB	Anti-Endothelin receptor B	ICC (1/500)	Chemicon (AB3284)
GD2	Anti-Human Diasilganglioside GD2	ICC, FC (1/100)	BD Pharmingen (554272)
Sox-2	Anti-Sox-2 (clone E-4)	ICC (1/50)	Santa Cruz (SC-365823)
Griffonia Simplicifolia lectin I. Specific for endothelial cells.	GSA I Lectin-Rhodamine	IHC (1/100)	Vector Labs (B-1105)
	Secondary antibodies	Application (dilution)	Company (Reference)
	Alexa Fluor®488 Donkey Anti-Mouse IgG (H+L)	ICC (1/1000), FC (1/200), IHC (1/500)	Thermo Fisher Scientific (A-21202)
	Alexa Fluor®568 Goat Anti-Mouse IgG (H+L)	ICC (1/1000)	Thermo Fisher Scientific (A-11004)
	Alexa Fluor®488 Goat Anti-Mouse IgM Heavy Chain	FC (1/200)	Thermo Fisher Scientific (A-21042)
	PE Rat Anti-Mouse IgG1	FC (1/200)	BD Pharmingen (550083)
	Biotinylated Goat Anti-Mouse IgG	IHC (1/2000)	Vector Labs (BA-9200)
	Alexa Fluor®488 Donkey Anti-Rabbit IgG (H+L)	ICC (1/1000), FC (1/200)	Thermo Fisher Scientific (A-21206)
	Alexa Fluor®568 Goat Anti-Rabbit IgG (H+L)	ICC (1/1000), IHC (1/500)	Thermo Fisher Scientific (A-11011)
	FITC Goat Anti-Rabbit IgG (H+L)	FC (1/200)	Jackson ImmunoResearch (111-096-144)
	PE Goat Anti-Rabbit IgG (H+L)	FC (1/200)	Jackson ImmunoResearch (111-116-144)
	Biotinylated Goat Anti-Rabbit IgG	IHC (1/2000)	Vector Labs (BA-1000)
	PE Donkey Anti-Goat IgG (H+L)	FC (1/200)	Jackson ImmunoResearch (705-116-147)

Supplementary Table 5: Primers used in RT-PCRs

mRNA	Sense	Sequence (5′–3′)
Bmi-1	Forward	ATTGTTCGTTACCTGGAGACC
	Reverse	GGCAGCATCAGCAGAAGG
Msi-1	Forward	CCCTGGCTACACCTACC
	Reverse	AGGCAGTGAGAGGAATGG
Oct-4	Forward	CTTGCTGCAGAAGTGGGTGGAGGAA
	Reverse	CTGCAGTGTGGGTTTCGGGCA
Acta-2	Forward	TTGGCTTGGCTTGTCAGG
	Reverse	GCTTTAGGGTCGCTGGAG
CD44	Forward	CTCATACCAGCCATCCAATG
	Reverse	GAGTCCATATCCATCCTTCTTC
Eng/CD105	Forward	CTTCCTCCTCCACTTCTAC
	Reverse	GGACTTCCTGGTCTTGAG
MEF2C	Forward	GCGAAAGTTCGGATTGATGAAGA
	Reverse	GTGGATGTCAGTGCTGGCGTA
S100a10	Forward	GGACCAGTGTAGAGATGGCAA
	Reverse	TCTTATCAGGGAGGAGCGAA
TGFB1	Forward	GCAGCACGTGGAGCTGTA
	Reverse	CAGCCGGTTGCTGAGGTA
Vim	Forward	AACTTCTCAGCATCACGATGAC
	Reverse	TTGTAGGAGTGTCGGTTGTTAAG
Gapdh	Forward	GTGAAGGTCGGAGTCAACG
	Reverse	TGAGGTCAATGAAGGGGTC
