# Supplementary material for: Neural crest derived progenitor cells contribute to tumor stroma and aggressiveness in stage 4/M neuroblastoma
Source: Oncotarget. 2017 Sep 21;8(52):89775–92. doi: 10.18632/oncotarget.21128 (PMC5685708; doi:10.18632/oncotarget.21128)
Supplement: Supplementary file 2 [file oncotarget-08-89775-s002.docx]

**Supplementary Table 3: Clinical information for samples included in TMAs.**

**TMA #1.**

| **BIOPSY NUMBER** | **TMA** | **SEX** | **AGE (months, at biopsy)** | **LAST CONTROL** | **CHEMOTHERAPY** | **LOCALIZATION** | **HISTOLOGY** | **PROGNOSIS** | **MKI** | **NMYC Amp.** | **METASTASIS** | **STAGE** | **RELAPSE/ PROGRESSION** | **EXITUS** |
| --- | --- | --- | --- | --- | --- | --- | --- | --- | --- | --- | --- | --- | --- | --- |
| 11B08897 | 1 | M | 72 | 20/03/14 | YES | A | POORLY DIFF. NB | POOR | L <2% | NO | YES | M | YES | YES |
| 11B10154 | 1 | W | 6 | 09/05/12 | NO | RP | POORLY DIFF. NB | POOR | H>4% | YES | YES | MS | NO | YES |
| 11B10662 | 1 | M | 6 | 15/01/13 | NO | RP | POORLY DIFF. NB | FAVORABLE | L <2% | NO | NO | L2 | NO | NO |
| 11B12879 | 1 | W | 12 | 02/10/13 | NO | A | POORLY DIFF. NB | FAVORABLE | I 2-4% | NO | NO | L2 | NO | NO |
| 11B15729 | 1 | M | 20 | 10/03/15 | NO | T/P | DIFF NB | FAVORABLE | L <2% | NO | NO | L2 | NO | NO |
| 11B17742 | 1 | M | 75 | 15/12/11 | YES | RP | DIFF NB | POOR | L <2% | YES | YES | M | YES | NO |
| 11B19126 | 1 | W | 68 | 05/02/13 | YES | A | DIFF NB | POOR | L <2% | NO | YES | M | YES | NO |
| 11B19376 | 1 | M | 9 | 15/01/13 | YES | RP | DIFF NB | ND | L <2% | NO | NO | L2 | NO | NO |
| 11B20185 | 1 | W | 10 | 09/05/12 | YES | RP | DIFF NB | FAVORABLE | L <2% | YES | YES | MS | ND | YES |
| 11B23937 | 1 | W | 56 | 13/01/14 | YES | T/P | DIFF NB | FAVORABLE | L <2% | NO | NO | L2 | NO | NO |
| 11B26365 | 1 | W | 59 | 24/09/14 | NO | T/P | GNB INTERMIXED | FAVORABLE | L <2% | NO | NO | L1 | NO | NO |
| 11B29912 | 1 | M | 27 | 05/02/12 | YES | T/P | DIFF NB | POOR | H>4% | NO | YES | M | NO | YES |
| 12B02911 | 1 | M | 29 | 26/03/15 | NO | A | UNDIFF NB | POOR | H>4% | NO | YES | M | YES | NO |
| 12B05856 | 1 | M | 17 | 18/03/15 | YES | A | DIFF NB | FAVORABLE | L <2% | NO | YES | M | NO | NO |
| 12B06600 | 1 | W | 6 | 03/04/14 | NO | T/P | DIFF NB | FAVORABLE | L <2% | NO | NO | L1 | NO | NO |
| 12B11265 | 1 | M | 32 | 26/03/15 | YES | A | DIFF NB | FAVORABLE | L <2% | NO | YES | M | YES | NO |
| 12B12989 | 1 | M | 60 | 01/10/12 | YES | T/P | UNDIFF NB | POOR | H>4% | YES | YES | M | YES | YES |
| 12B14969 | 1 | W | 10 | 02/02/15 | NO | RP | UNDIFF NB | POOR | H>4% | YES | NO | L2 | NO | NO |

| Biopsies with the same color belong to the same patient. M, man; W, woman; RP, retroperitoneal; A, adrenal; T/P, thoracic/paraspinal; C, cervical; UNDIFF, undifferentiated; DIFF, differentiating; NB, neuroblastoma; GNB, ganglioneuroblastoma; MKI, Mitosis-karyorrhexis index; H, high; I, Intermediate; L, low; ND, not determined. |
| --- |
|  |

**TMA #2.**

| **BIOPSY NUMBER** | **TMA** | **SEX** | **AGE (Months, at biopsy)** | **LAST CONTROL** | **CHEMOTHERAPY** | **LOCALIZATION** | **HISTOLOGY** | **PROGNOSIS** | **MKI** | **NMYC Amp.** | **METASTASIS** | **STAGE** | **RELAPSE/ PROGRESSION** | **EXITUS** |
| --- | --- | --- | --- | --- | --- | --- | --- | --- | --- | --- | --- | --- | --- | --- |
| 12B23746 | 2 | W | 14 | 02/02/15 | YES | A | DIFF NB | FAVORABLE | L <2% | YES | NO | L2 | NO | NO |
| 13B07988 | 2 | M | 10 | 16/05/13 | YES | A | GNB INTERMIXED | FAVORABLE | L <2% | YES | YES | MS | NO | NO |
| 13B09218 | 2 | M | 45 | 20/03/15 | NO | C | POORLY DIFF. NB | POOR | H>4% | NO | YES | M | NO | NO |
| 13B11807 | 2 | M | 76 | 29/01/15 | YES | A | GNB NODULAR | POOR | L <2% | NO | NO | L2 | NO | NO |
| 13B12918 | 2 | M | 20 | 11/06/15 | YES | T/P | DIFF NB | FAVORABLE | I 2-4% | NO | NO | L2 | NO | NO |
| 13B13204 | 2 | M | 83 | 20/08/14 | NO | RP | UNDIFF NB | POOR | I 2-4% | YES | YES | M | YES | YES |
| 13B18622 | 2 | M | 33 | 12/02/14 | YES | RP | GNB INTERMIXED | FAVORABLE | L <2% | NO | YES | M | YES | YES |
| 13B18799 | 2 | M | 48 | 20/03/15 | YES | C | GNB INTERMIXED | FAVORABLE | L <2% | NO | YES | M | NO | NO |
| 13B22436 | 2 | M | 87 | 20/08/14 | YES | A | UNDIFF NB | POOR | L <2% | YES | YES | M | YES | YES |
| 13B23949 | 2 | W | 29 | 09/04/14 | YES | A | DIFF NB | ND | L <2% | NO | YES | M | YES | YES |
| 13B30314 | 2 | M | 46 | 14/03/15 | NO | A | GNB NODULAR | POOR | H>4% | NO | YES | M | NO | NO |
| 14B11475 | 2 | W | 70 | 25/03/15 | NO | RP | POORLY DIFF. NB | POOR | I 2-4% | NO | YES | M | NO | NO |
| 14B13182 | 2 | M | 37 | 13/03/15 | YES | A | DIFF NB | ND | L <2% | NO | YES | M | NO | NO |
| 14B16172 | 2 | M | 96 | 20/08/14 | YES | A | UNDIFF NB | ND | ND | YES | YES | M | YES | YES |
| 14B16722 | 2 | W | 15 | 31/12/14 | YES | T/P | POORLY DIFF. NB | ND | ND | YES | NO | L2 | NO | NO |
| 14B20854 | 2 | W | 74 | 22/06/15 | YES | A | POORLY DIFF. NB | POOR | L <2% | NO | YES | M | NO | NO |
| 14B24936 | 2 | W | 2 | 12/10/14 | NO | A | POORLY DIFF. NB | FAVORABLE | L <2% | NO | YES | MS | ND | YES |

| Biopsies with the same color belong to the same patient. M, man; W, woman; RP, retroperitoneal; A, adrenal; T/P, thoracic/paraspinal; C, cervical; UNDIFF, undifferentiated; DIFF, differentiated; NB, neuroblastoma; GNB, ganglioneuroblastoma; MKI, Mitosis-karyorrhexis index; H, high; I, Intermediate; L, low; ND, not determined. |
| --- |
